# Contrasting modes of cultural evolution: Kra-Dai languages and weaving technologies

**DOI:** 10.1017/ehs.2025.10008

**Published:** 2025-07-25

**Authors:** Christopher D. Buckley, Emma Kopp, Thomas Pellard, Robin J. Ryder, Guillaume Jacques

**Affiliations:** 1 Tracing Patterns Foundation, Berkeley, CA, USA; 2 CEREMADE, CNRS, Université Paris-Dauphine, PSL University, Paris, France; 3 CRLAO (EHESS, CNRS, Inalco), Paris, France; 4 Department of Mathematics, Imperial College London, London, UK; 5 École Pratique des Hautes Études, PSL University, Paris, France

**Keywords:** Kra-Dai, Tai-Kadai, language, weaving, looms, cultural evolution, punctuated equilibrium, phylolinguistics

## Abstract

We investigate and compare the evolution of two aspects of culture, languages and weaving technologies, amongst the Kra-Dai (Tai-Kadai) peoples of southwest China and Southeast Asia, using Bayesian Markov-Chain Monte Carlo methods to uncover phylogenies. The results show that languages and looms evolved in related but different ways and bring some new insights into the spread of the Kra-Dai speakers across Southeast Asia. We found that the languages and looms used by Hlai speakers of Hainan are outgroups in both linguistic and loom phylogenies and that the looms used by speakers of closely related languages tend to belong to similar types. However, we also found differences at a deep level both in the details of the evolution of looms and languages and in their overall patterns of change, and we discuss possible reasons for this.

## Graphical abstract

**Figure:**
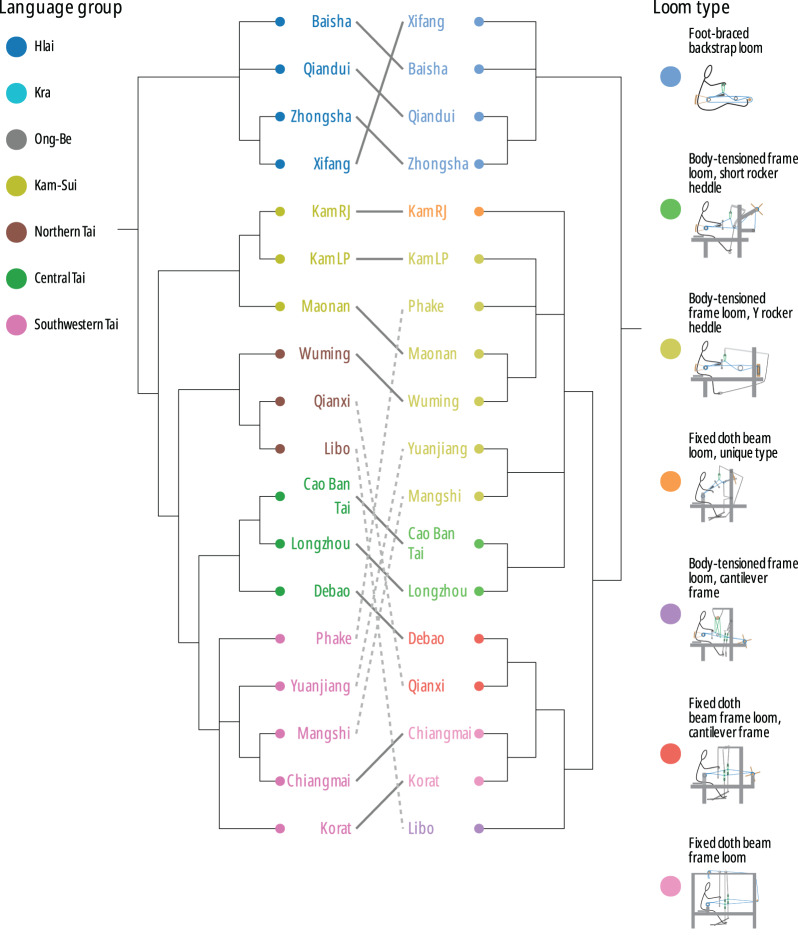

## Short summary

The paper compares the evolutionary histories of the weaving looms and languages of the Kra-Dai (Tai-Kadai) peoples, who live in southern China and Southeast Asia. Using datasets of the features of looms and languages, the authors constructed phylogenies of both and compared them. The phylogenies are broadly comparable, reflecting the migrations and divergences of the Kra-Dai since the Neolithic period. There are differences, however: archaic looms found near the borders between China, Myanmar, and Assam hint at early migration events or shifts in looms or languages. The results also show that languages and technologies evolve in fundamentally different ways: Languages undergo smooth change and technologies evolve in bursts interspersed with periods of stasis.

## 1. Introduction

Both languages and weaving methods are complex traditions that are passed down from generation to generation, with modifications. Over time, modifications accumulate, and traditions evolve: this fact enables researchers to investigate their histories using tree models. In linguistics, tree-like representations of language change have been used for nearly two centuries (Pellard et al., 2026), and in recent years, phylogenetic methods, particularly using Bayesian approaches, are becoming a mainstream technique for inferring language history (Gray & Atkinson, 2003, Gray et al., 2009, Chang et al., 2015, Kolipakam et al., 2018, Sagart et al., 2019, Heggarty et al., 2023). Phylogenetic methods have similarly been applied to other aspects of culture (Mace & Holden, 1985), including material culture and technologies. The methods used include distance-based methods (Jordan & Shennan, 2003, Saslis-Lagoudakis et al., 2014) and maximum parsimony (Buchanan & Collard, 2007, Tehrani et al., 2010, Le Bomin et al., 2016, O’Brien et al., 2016) and, to a lesser extent, Bayesian methods (Matthews et al., 2011, Buckley & Boudot, 2017, Learmouth et al., 2024).

Several studies have examined the similarities and differences between artfact and linguistic evolution. Jordan & O’Neill (2010) found correlations between longhouse architecture and languages on the Northwest coast of America, while Jordan & Shennan (2003) found that basketry traditions and languages in California evolved differently. Passmore et al. (2024) uncovered correlations between linguistic and music diversity at local scales, but few at the global scale. Learmouth et al. (2024) compared Pama-Nyungan languages and cultural practices in Australia and found correlations between languages and initiation rites but not between languages and mortuary rituals or petroglyphs. Similarly, Brown et al. (2014) found that music and language evolved differently amongst Indigenous Taiwan populations. The results of these comparisons show that language and material culture are sometimes correlated, especially where they follow the same cultural boundaries, but that they do not necessarily evolve in tandem.

These observations prompt the broader question of whether language and other cultural characteristics evolve in similar ways (both in principle and in practice), and to what extent we should expect them to be correlated. Language is the main identifier used to define ‘ethnolinguistic’ groupings (as the name implies), whereas material and social aspects of human culture are not usually incorporated in such definitions. Is language a valid proxy for other aspects of culture in evolutionary studies, and what might the limitations of this approach be?

In this study, we address this issue by making a comparison between the patterns of evolution of the languages and weaving technologies amongst the Kra-Dai peoples of East Asia and Southeast Asia. We choose weaving technologies because (like languages) their core features are conservatively transmitted, generally from parent (or close relative) to child within rural societies. They are rich in terms of complexity and variation (like languages), and they offer (in principle at least) considerable scope for innovation. As with core linguistic vocabulary, some loom technologies have considerable time-depths, comparable to that of languages (Buckley & Boudot, 2017).

### 1.1. Weaving among Kra-Dai-speaking peoples

The Kra-Dai are one of several groups who are distributed across the southern provinces of China and several countries of mainland Southeast Asia, including Vietnam, Laos, Thailand, and Myanmar, as well as Assam province in northeast India. They share their territories with speakers of languages from other major families, including Austroasiatic, Sino-Tibetan, Hmong-Mien, and Austronesian.

Weaving is an important part of the culture of Kra-Dai people and their neighbours, both for practical reasons (making useful items such as clothing, bed coverings, and mosquito nets) and for the expression of personal identity and status. Though many peoples weave, unlike language, there is no absolute necessity to do so. Some groups do no weaving and obtain cloth by trading with near neighbours. Some weave for their own use, and some do so for their own use and for localised trade. Most weavers use a single loom for weaving broad cloth, but in a few cases, two different looms are used: One for plain cloth, and one for patterned cloth (Buckley & Boudot, 2017). Many weavers also use a simple loom for weaving narrow bands (for making belts and straps) in addition to their main loom: These looms are probably little changed from the earliest looms employed in the region (Buckley, 2023).

Fashions for designs and colours may circulate amongst neighbouring peoples as a result of trade and exposure to designs at local markets and festivals, but loom technology usually stays within the family or close kin group. In this respect, there is a basic resemblance between the transmission of language and looms. There are also some differences: loom technology is mainly transmitted from mother to daughter (or aunt to niece) and tends to stay within the female line.

**Figure 1.**
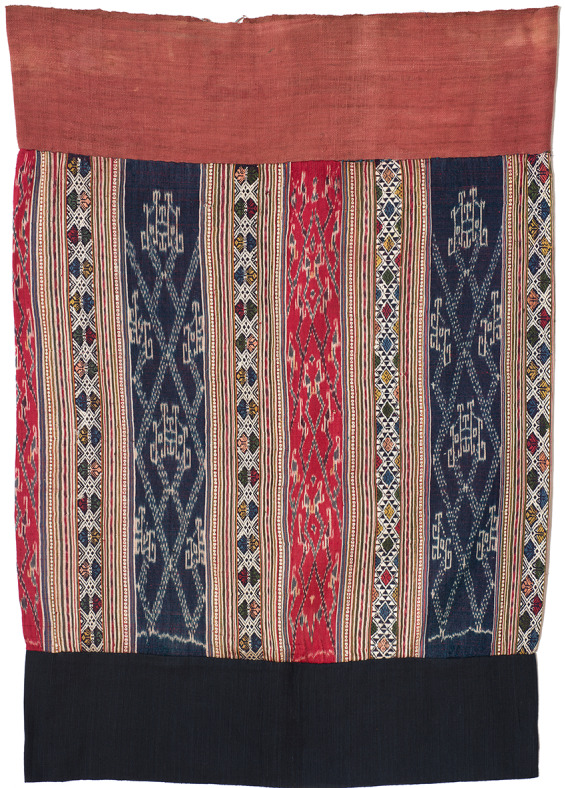
Ceremonial skirt woven from silk by a Tai weaver near the northern Laos–Vietnam border, using discontinuous and continuous supplementary weft and ikat patterning techniques, 65cm × 91cm (Tracing Patterns Foundation collection).

Many Kra-Dai speakers are particularly adept weavers: in northern Vietnam and Laos (for example), the fine silk ceremonial cloths that they make circulate via trade among Austroasiatic speaking neighbours as well as fellow Kra-Dai (Figure 1). The weaving technologies used by the Kra-Dai people range from very simple looms used by Hlai speakers on the island of Hainan (as their main loom), to complex frame looms used on the mainland (Buckley, 2023). Some of the latter incorporate sophisticated pattern-recording systems that encode the designs to be woven in a permanent form.

### 1.2. Weaving, farming, and language families

#### 1.2.1. The emergence and spread of weaving

The earliest clear archaeological traces of textile production in East Asia are spindle whorls (used for spinning yarn) made of stone and pottery. These implements first appear between 9000 and 7500 BP, possibly independently, in early millet-farming sites of the northeast, such as Xinglongwa (兴隆洼), Mid-Yellow River Basin sites such as Peiligang (裴李岗) and Jiahu (贾湖) (Smith & Lee, 2019), as well as rice-farming Lower Yangtze River Basin at Kuahuqiao (跨湖桥) (Rao, 2019, 48–53) and Jingtoushan (井头山) (Sun et al., 2021)

From these core areas, spindle whorls gradually spread south and west. By 4900–4100 BP, spindle whorls are present at archaeological sites across southern China such as Yunglong (涌浪) in Hong Kong (Lu, 1997–1998), subsequently appearing in Phúng Nguyên sites in northern Vietnam from around 4000 BP (Bellwood, 2005, 131–132). Their presence charts the production of yarn in significant quantities and, by implication, the spread of weaving, alongside rice agriculture and Neolithic lifeways.

Aside from spindle whorls, the earliest traces of weaving technologies are wooden and bone tools, including weft beaters and yarn insertion tools, excavated from the Hemudu (河姆渡) culture site at Tianluoshan (田螺山) near the mouth of the Yangtze, dating from around the same time that spindle whorls make their first appearance (Sun et al., 2007). There is insufficient evidence to be sure of the precise forms of the looms on which these tools were employed, but they were probably simple, body-tensioned devices. The first loom remains that are clearly interpretable consist of jade components from a female Liangzhu (良渚) culture burial at the nearby site of Yuhang (余杭) from around 4500 BP (Buckley, 2023, 156). This loom is clearly reconstructable as a foot-braced body tensioned loom similar to those still in use by Li people (Hlai speakers) on Hainan island today and is included in the group of looms in our study. Foot-braced backstrap looms are not restricted to South-East China and are archaeologically in sites on the Tibetan plateau in Yunnan, dated 2350–2100 cal. BP (Hao et al., 2024).

The earliest evidence for more complex looms with frames comes from Jing’an (靖安) in Jiangxi province, where wooden loom parts were retrieved from tombs dating from the Eastern Zhou period (779–221 BCE), along with textile fragments (Zhao et al., 2012, Boudot & Buckley, 2015, 31). These looms were body-tensioned looms similar to looms 2–6 in Figure 3. The designs of these looms betray their origins, since in essence they consist of simple body-tensioned looms transposed into frames, preserving many of the features of older designs. These looms allowed the production of longer, wider cloths in a more efficient and reproducible fashion. Tomb engravings show that at least two types were in use domestically by the Han dynasty (202 BCE–220 CE), corresponding to looms 2 and 3, versus 4 and 5 in Figure 3. Archaeological remains reveal that the fibres used by these early weavers included bast fibres such as ramie (*Boehmeria nivea*), hemp (*Cannabis sativa*), kudzu vine (*Pueraria lobata*), and silk (Huang & Chen, 2002, Zhao, 2014, Gong et al., 2016, Liu et al., 2017).

A major technological development seems to have begun around two millennia ago with the appearance of looms with fixed cloth beams and novel systems for making openings in the warp. These looms have been gradually replacing older, body-tensioned designs, a process that is still continuing today. The area that is now southern China was a key centre for many of these innovations. Based on their present-day weaving practices and looms, the Kra-Dai appear to have played a central role in many of these new technologies, particularly in the development of pattern-recording systems, which remain the (almost) exclusive preserve of Kra-Dai speakers on the Asian mainland (Buckley, 2023).

#### 1.2.2. Farming and languages

Rice and millet farming is not only correlated with the development of weaving as described above but also to the spread of language families through demic diffusion (Bellwood, 2005), a model that is applicable to all the major language families of East Asia, including Sino-Tibetan, Austroasiatic, Austronesian, Hmong-Mien, and Kra-Dai. The spread of Sino-Tibetan, for example, is associated with demic diffusion related to the domestication of broomcorn and foxtail millets in the Middle Yellow River basin in the Early Neolithic period (Sagart, 2008, Stevens & Fuller, 2017, Sagart et al., 2019, Zhang et al., 2019, Wang et al., 2021), while that of Austroasiatic is associated with the spread of rice agriculture from the Mid-Yangtze River basin (Peiros & Shnirelman, 1998, Bellwood, 2005). Kra-Dai and Austronesian language speakers are mainly wet rice farmers, and a genetic relationship between these two families is increasingly becoming the consensus view (Sagart, 2004, Ostapirat, 2005, Norquest, 2013).

The correspondence between linguistic and archaeological data regarding Kra-Dai and Austronesian remains a matter of debate: Sagart (2008, 2022) argues that these two families are related to Sino-Tibetan, and that both originate from the Lower Yellow River cultures of Houli (后李) and perhaps later Dawenkou (大汶口), whereas Tao et al. (2023) link their predecessors to the Neolithic cultures of the Lower Yangtze and coastal regions. Whether the homeland of the Kra-Dai lies in the Mid-Yellow River or the Lower Yangtze area, there can be no doubt that the common ancestors of all Kra-Dai speaking people knew weaving, as confirmed by the reconstructibility of a verb for ‘weave’ to the common ancestor of all of these languages (Proto-Tai *tam B, Proto-Kam-Sui *tam^3^, Buyang *tam*^54^; Thurgood 1988, Li 2000, Pittayaporn 2009). The same is true of other major language families of East Asia, such as Sino-Tibetan (Jacques et al., 2025) and Austro-Asiatic.

In summary, there is a correlation between the emergence and spread of language families, agriculture, and evidence of weaving. These are aspects of novel, sedentary lifeways that spread across the region, accompanied by demographic shifts and population expansions. Associations between emerging language groups (such as the Kra-Dai) and archaeological cultures are difficult to discern, so reconstructing phylogenetic histories provides a perspective that is complementary to that of the archaeological record.

## 2. Data and methods

### 2.1. Linguistic dataset

Our Kra-Dai languages dataset is based on that of Tao et al. (2023), a database of basic vocabulary comprising lexical forms for 90 concepts in 100 languages, annotated for cognacy, with a total of 647 cognate sets, encoded as a matrix of binary presence/absence traits. The coverage of this dataset for languages within the borders of China (where the greatest diversity of Kra-Dai languages is found) is comprehensive, while that of languages outside this region less so.

We augmented this dataset with data on two languages of Assam: Tai Phake (Morey, 2007) and Ahom (Morey et al., N.d). The only extinct (‘fossil’) language included in our dataset, Ahom, is known from written documents from the Ahom kingdom, which was founded in 1228 in Assam by a prince named Sukapha, centred in what is now Ruili (瑞丽) in Yunnan province, and which lasted until 1826 (Morey, 2004, 207). In the early part of the 19th century, the Ahom people had already assimilated to local Assamese culture, and their language had ceased to be spoken, though transmission of the script continued. Our source (Morey et al., N.d) is based in part on a native wordlist (the *Bar Amra*) from the 18th century, augmented with textual examples from manuscripts from the 14th to the 18th centuries.

The Ahom script lacks tone marks and presents spelling variants, some of which are purely graphic, others such as the alternation between *n* and *l* may reflect dialectal variants. These philological difficulties did not impede the identification of the Proto-Tai etymon (from Pittayaporn, 2009) for the Ahom forms in the list: As a Southwestern Tai (SWT) language, cognate coding was considerably easier than Kra and Hlai languages, whose historical phonology involves more sound changes and whose cognates are less obvious. Works such as those by Ostapirat (2000), Norquest (2015), and Chen (2018) clarify the sound correspondences. Even though no reconstruction of Proto-Kra-Dai is yet available, no significant difficulties were encountered in assembling the list of 90 concepts used in this study.

In the course of assembling the linguistic dataset, we corrected some apparent errors in the cognate coding regarding Kra languages, where superficially similar but unrelated etyma had been coded as cognates, even though the sound correspondences do not fit with existing understanding (Ostapirat, 2000). These corrections increased the number of cognate sets to 653.

The 90 concepts have been further classified into four groups based on part of speech: verbs, nouns, adjectives, and others (pronouns, negation, and numerals). While the applicability of these categories to isolating languages of the Kra-Dai family has been disputed,^1^ there are arguments for positing adjectives as distinct from verbs and nouns in the Tai branch (Post, 2008).

### 2.2. Weaving technologies dataset

Our Kra-Dai weaving technologies (looms) dataset is based on that of Buckley & Boudot (2017). It includes 21 looms from that dataset, with new data on eight Kra-Dai looms added, mainly from northern Vietnam and neighbouring regions. The data is based upon museum specimens and published sources. As with languages, it records the presence/absence of a set of characters; in this case, these are functional attributes such as warp beam, cloth beam, weft beater, and so on. The attributes and their descriptions, together with the sources we used, are available in the online supplementary materials.

The looms dataset contains fewer taxa than the languages dataset but has fair coverage of Tai/Dai and Kam-Sui looms. There are no examples of looms from Kra or Ong-Be speakers, since we have not been able to obtain sufficient information from these groups. What little we know suggests that these groups have weaving traditions that are Sinicised and retain no older characteristics.

For both languages and looms, we have chosen datasets that cover the diversity of types and geographic regions, with broadly similar and comparable coverage for both. We include a slightly higher proportion of looms from northern Vietnam, since this area is a hotspot for loom diversity, even though the linguistic diversity in this area is less than in neighbouring Chinese provinces. From the point of view of understanding evolutionary patterns, it is important for our datasets to accurately represent diversity.

The key requirements for the comparison of loom and language evolution are firstly to have sufficient coverage in both datasets to adequately resolve phylogenies and secondly to have sufficient directly comparable datapoints across both datasets. We address these points in the discussion of the results below. There is sufficient overlap between the datasets to be able to make meaningful comparisons and to test for similarities in phylogenies quantitatively.

The patterning systems used by some Kra-Dai weavers are detachable from their looms and can be transferred from one loom to another. This means that the looms and patterning systems may evolve independently of each other to some degree. To investigate this aspect, we specified for each feature whether it relates to the basic loom structure (159 traits) or patterning (27 simple patterning and 30 complex patterning traits).

### 2.3. Phylogenetic models

We inferred phylogenies for both languages and looms using a Bayesian approach, employing Markov Chain Monte Carlo methods to explore posterior distributions of phylogenetic trees fitting the data. We tested a variety of models, and as far as possible we applied the same models to both languages and looms. There are some necessary differences, for example, the incorporation of tree priors for the languages that capture historical data. These are discussed in detail below.

We modelled all of our data using a tree prior, a clock model, and a substitution model. The tree prior consists of our assumptions concerning the topologies and branch lengths of output trees, while the clock model links the length of each branch to the number of changes on that branch. The substitution model constrains the ways in which a given character may change (mutate) from one state to another.

For our tree prior, we chose the fossilised birth–death model, which is an extension of the birth–death model (Gavryushkina et al., 2014) that allows for fossil taxa (i.e. taxa that have disappeared). It is often used to model the evolution of lineages at the level of individual taxa.

For the clock model, we tested both a strict model, which assumes that all substitutions in the tree happen at the same rate across branches, and a relaxed clock model (Drummond et al., 2006), which allows evolutionary rates to vary between branches.

As regards the substitution model, the simplest type is the continuous-time Markov Chain (CTMC) model (Gray & Atkinson, 2003, Bouckaert et al., 2012), where each character can be either absent or present and may change according to a rate matrix that is invariant with time. In our study, however, we use the binary covarion model, which allows a degree of flexibility in mutation rates, which may switch between ‘fast’ and ‘slow’ conditions (Tuffley & Steel, 1998, Penny et al., 2001); this model is widely used to model cognate data (Hoffmann et al., 2021). For the languages data, we tested models with a homogeneous mutation rate matrix across traits, and we also tested a model that partitioned traits according to their parts of speech, into four categories (adjectives, nouns, verbs, and other words) and allowed a heterogeneous mutation rate that varies between these four categories. For the looms data, we partitioned the data into different classes (Levels), described below, and tested a model that allowed these to mutate at different rates.

### 2.4. Assessing goodness-of-fit

A Bayesian phylogenetic analysis yields a set of trees sampled from the posterior distribution: For each analysis, these were summarised by computing a majority-rule consensus tree, which shows nodes that are present in 50% or more of the sampled trees. To assess relative goodness-of-fit, we computed the marginal likelihood (ML) of each model. The output presented in the main text corresponds to the model with best ML. The analyses were carried out using the BEAST software (Bouckaert et al., 2019); details can be found in the Supplementary Information.

Aside from ML scores, in assessing the results we looked at consistency of support for key nodes in tree topologies and robustness to change of models.

### 2.5. Priors for linguistic analysis

Unlike Tao et al. (2023), who set eight age priors, the only constraints we applied were to set the date of the common ancestor of SWT to lie between 700 and 1000 CE, and the date of Ahom to lie between 1300 and 1800 CE. The date range for the emergence of SWT follows Pittayaporn (2014), who provides two pieces of evidence showing that the latest layer of Chinese borrowings in Proto-SWT corresponds to Late Middle Chinese (LMC, post 700 AD) rather than Early Middle Chinese (EMC). In this later layer, plain EMC voiced stops *b*, *d*, *g* correspond to aspirated stops in Proto-SWT and EMC palatalised labials *bj-*, *pj-* to labiodentals (Table 1), though the correspondences suggest several sub-layers: examples with EMC *pj-* to aspirated *p^h^- in Proto-SWT indicate an intermediate stage between *pj- and *f- in the donor language. On the other hand, there is no evidence of Early Mandarin (14th century) loanwords into Proto-SWT. This date is also in line with historical evidence (Evans, 2016). In particular, the ethnonym Shan (Syam), used for various SWT-speaking groups, is attested in Cham and Pagan inscriptions from the 11th century (Luce, 1985).

**Table 1.** Some representative examples of the devoicing of voiced stops and development of labiovelars in the latest layer of Chinese loanwords in SWT (the EMC and LMC forms are from Baxter (1992) and Pulleyblank (1991), respectively)

Proto-SWT	EMC	LMC
*t^h^ue^B^ ‘bean’	豆 *duwH*	*thew‘*
*k^h^we^A^ ‘eggplant’	茄 *gja*	*khia*
*p^h^ue^B^ ‘person’	甫 *pjuX*	*fuě’*
*vu:^A^ ‘float’	浮 *bjuw*	*fhuw*

Tao et al. (2023) used other temporal priors, which are more speculative and therefore were not enforced in this study. Since we enforce only a small number of age constraints (one internal node and one fossil leaf), the posterior distribution of all unconstrained ages can be expected to have a higher variance. These ages are not the focus of our analysis, and this extra variance has no bearing on our main results.

### 2.6. Interdependency in loom traits

**Figure 2.**
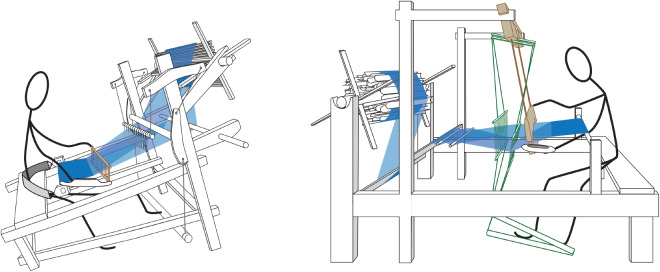
Comparison of reeds (shown in brown colour) of a simple kind on a Cao Ban Tai loom (left) and on a Zhuang cantilever loom (right). The cantilever frame on the Zhuang loom allows the suspension of a heavier, swinging reed that can be used as a weft beater and a device for organising warps. Together, these components form a ‘module’ that occurs in many looms with similar frames.

Languages and looms differ in one important respect: While it is standard to model cognate linguistic forms as varying independently, this modelling assumption can be questioned for certain loom traits that are dependent upon others. This is a general characteristic of technologies, where components build upon (and sometimes depend upon) the presence of other components. In some cases, the dependency is straightforward, for example, the presence of a handle on a weft-beater (and its associated character) is dependent on the presence of a weft-beater (and its associated character). Some kinds of interdependency between loom characters are more complex. For example, some looms incorporate a reed, a comb-like device that separates warp yarns and helps to keep them in order. In its simplest form, the reed is a lightweight component that floats in the warp yarns. Adding overhead beams to the loom to make a cantilever frame allows the reed to be suspended in front of the weaver, which then allows the use of a heavier reed that can also be used to beat in the weft. There are other ways to suspend a reed: it can be suspended from two curved bamboo struts, or it can be attached to a bar that pivots at the base of the loom, but the cantilever frame is particularly effective in this regard. Other components can also be attached to the cantilever, such as a pair of linked heddles. Whatever the original reason for the cantilever feature, its presence opened up a range of new design possibilities which were exploited by weavers in various ways (Figure 2), and in most cases, it is associated with a suspended reed.

Character interdependency can bias the inference of phylogenies by giving undue weight to the absence of characters at the bottom of the hierarchy. This is sometimes referred to as Maddison’s red/blue tail problem, following Maddison (1993). Various solutions have been proposed in the systematic biology literature (Brazeau et al., 2019, Hopkins & St. John, 2021), but it is typically more difficult to handle hierarchies with several levels.

To investigate whether this feature of character interdependency influences the outcome of the phylogenetic inference, we assigned ‘levels’ to the loom characters. Level 1 consists of all the characters that are independent of each other. Level 2 consists of characters that are sub-categories of Level 1 characters, and so on. Some Level 1 characters are independent of each other and of all other characters, for example, several kinds of sticks can be independently inserted into the warp as aids for making openings. Other Level 1 characters open up possibilities for further novel loom features. For example, a rigid horizontal frame linking the warp beam and cloth beam supports is a Level 1 character. Other features that are directly built upon this frame, such as upright members at the front and back, are Level 2 characters, while additional cross-pieces across the top of the loom are assigned to Level 3. We found that four levels were necessary to assign all of the loom characteristics in our dataset. We analysed Level 1 characters separately, then compared this with analyses of all four levels with various weightings applied.

## 3. Results

Convergence was verified for all the models tested, as described in the Supplementary Information. Overall, we found that the consensus trees that we obtained for both looms and languages varied little between models. We illustrate the results with consensus trees for both datasets that use the binary covarion model with relaxed clock and heterogeneous mutation rates. Trees for alternate models can be found in the Supplementary Information.

We first review some unique features that emerge from our analyses of the loom dataset: the modular nature of loom design, the dependencies between traits, and the rates of change of traits. We then describe the results of the phylogenetic analyses of both looms and languages.

### 3.1. Modular organisation in looms

**Figure 3.**
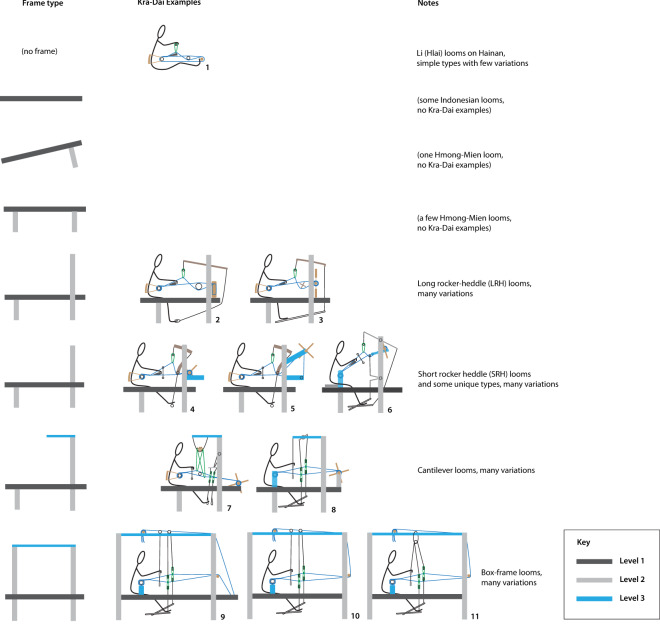
Illustration of how loom frame types (left) are built up in a modular fashion from basic components. These modules have been exploited by weavers to attach further devices, as shown in the examples of actual looms in the centre of the figure. With the exception of the Tai Libo loom (7), patterning features are not shown, in order to render the key structural features of the frames more clearly. As one moves down the figure from ‘no frame’ to increasingly complex designs, the possibilities opened up by the frames become greater and the complexity and variety of the looms increase. Key: 1: Hlai, 2: Kam LP, 3: Tai Phake, 4: Cao Ban Tai, 5: Tai Longzhou, 6: Kam RJ, 7: Tai Libo, 8: Tai Debao, 9: Tai Chiangmai, 10: Tai Xam Nuea, 11: Tai Korat.

In the process of assigning levels to the various features of looms in our study, we found that some features are the basis for entire suites of features that tend to co-occur (modules). This modular aspect of loom design is illustrated for some of the Kra-Dai looms in this study in Figure 3, which shows how groups of different loom designs are built upon the foundation of a rigid, horizontal frame. Many looms share similar basic frame features, with minor variations.

Similarly, certain functional features that work well together tend to co-occur as modules. The simplest system for making openings in the warp for weft insertion (known as shed and counter-shed in weaving terminology) consists of a rod that retains one opening and a unidirectional heddle for the complementary opening. This system requires the weaver to continually adjust the tension in the warp, and it works well in the case of the simplest (and oldest, based upon archaeological remains) body-tensioned looms. It is less effective in looms with a fixed cloth beam, since the weaver cannot alter the warp tension in this type of loom. For fixed-cloth beam looms, a different module for making warp openings is usually found, consisting of a pair of linked, bidirectional heddles worked by foot treadles, usually suspended from overhead bars (cantilever or box frame design).

### 3.2. Dependencies between traits in the loom phylogeny

To gauge the possible influence of trait interdependency on our results, we conducted three separate analyses of loom traits, assigning different weightings to each level. In the first analysis (Figure S1), we only included Level 1 traits, which are considered to be fully independent from each other. The resulting majority-rule consensus tree comprises three main clades: Simple foot-braced backstrap looms, body-tensioned frame looms (including types with heddles attached to long Y-shaped rockers and short rockers), and fixed cloth beam frame looms, the latter including the most complex types. The recent branches of this tree are poorly resolved, which is to be expected since we have discarded the portion of the data (Levels 2, 3, and 4), which describes the evolutionary elaboration of the basic features of the looms.

In the second analysis (Figure 7 and S2), each trait was assigned the same weight (i.e. no weighting), regardless of level. The consensus tree obtained has an overall topology similar to that of Level 1 analysis, but the addition of Levels 2, 3, and 4 provides better resolution, particularly for the fixed cloth beam frame looms, which incorporate refinements corresponding to the exploitation of the possibilities opened up by the presence of overhead beams (cantilever and box-frame looms).

In the third analysis (Figure S3), we applied a weighting that is inversely proportional to the degree of dependency: Level 1 has weight 8, Level 2 weight 4, Level 3 weight 2, and Level 4 weight 1. The consensus tree from this analysis has a very similar topology to the unweighted version in Figure 7, with two differences: The Tengchong loom appears among body-tensioned frame looms in the second analysis, whereas it is the outgroup of fixed cloth beam looms in the third analysis, and the cantilever frame fixed cloth beam looms occur in one clade in the second analysis, whereas they are a nested outgroup of the Tai fixed cloth beam frame looms in the third analysis.

These analyses show that all four levels are necessary for a full resolution of the phylogenetic tree, but that applying different weightings to the levels has a limited influence on the tree topology.

### 3.3. Rates of change of loom traits

**Figure 4.**
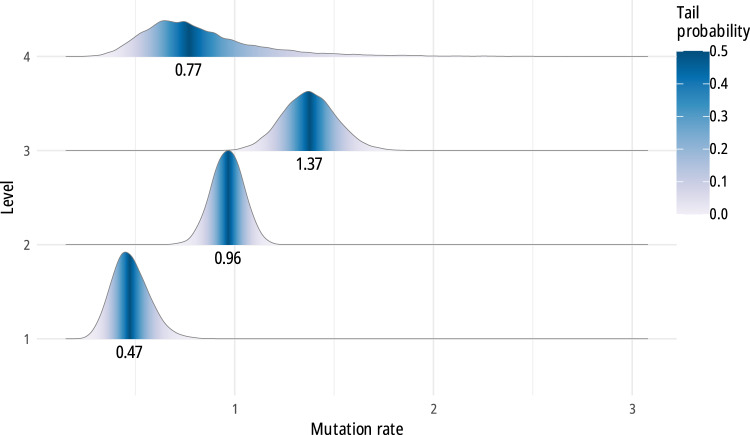
Probability density function and median values of the distribution of mutation rates (i.e. rates of change) in looms, by trait level (binary covarion, relaxed clock, heterogeneous rates, no weighting). Traits at Level 1 represent the most basic features of looms, some of which are the basis for other traits at Level 2 and so on. Traits at lower levels (mostly) evolve more slowly than traits at higher levels.

Coding the loom features by level also allowed us to perform a separate analysis allowing four distinct mutation rates, one for each of the levels of dependency. The resulting consensus phylogeny has the same topology as in the previous analyses, confirming that this aspect is robust as regards the details of the evolutionary model. We nevertheless uncovered significant differences in rates of change between the four levels. As shown in Figure 4 (see also Table S2), Level 1 traits, which are assumed to be independent of each other and correspond to basic features of looms, have the lowest mutation rate, Level 2 traits have a higher rate, and Level 3 traits have the highest one. For Level 4 traits, the evidence is less clear, and the mutation rate has a larger range of variation, probably due to the small number of characters (13 characters for Level 4, in comparison with 102 for Level 2).

### 3.4. Basic loom features vs. patterning features

We also performed two separate analyses using only basic loom features on the one hand, and patterning features (simple and complex) on the other hand. The topology obtained with basic traits only (Figure S4) is almost identical to the phylogeny with unweighted characters (Figure 7), with the exception of the position of the Tengchong loom. By contrast, the topology of the phylogeny obtained exclusively with patterning traits (Figure S5) has less structure, as shown by the low posterior probabilities of most groups, and the fact that, with a few exceptions, very few clades correspond to groups related either from the point of view of their loom type and linguistic subgroup.

These results suggest that basic loom features contain more phylogenetic signal than patterning features. This seems to be due in part to the fact that patterning features are more freely exchanged between looms, and in part to the fact that some of these features are optional: not all looms possess complex patterning systems, for example.

### 3.5. The linguistic phylogeny and dating of key nodes

**Figure 5.**
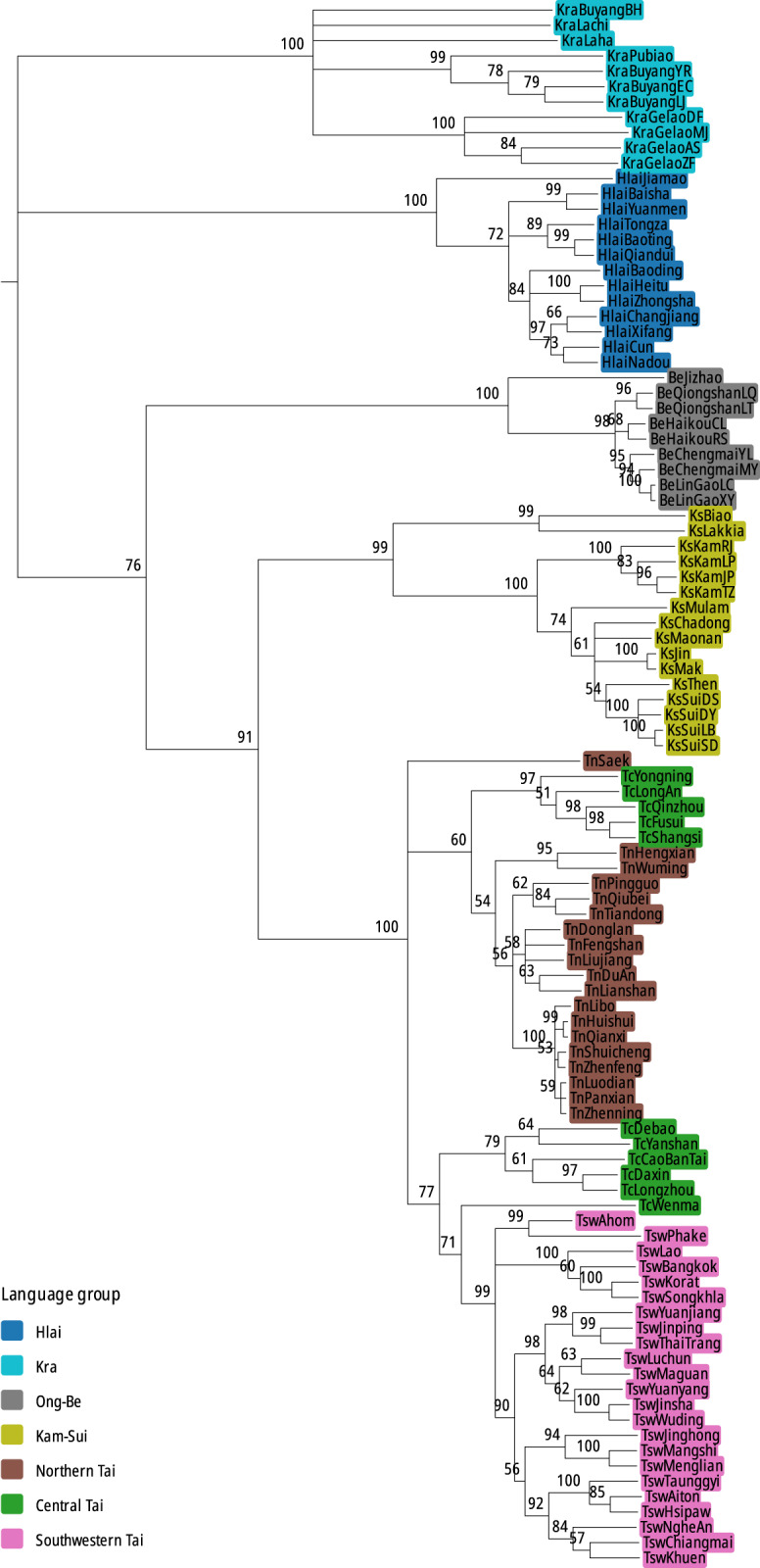
Majority-rule consensus tree for the languages (binary covarion, relaxed clock, heterogeneous rate by part-of-speech); each node is annotated for its posterior probability (in per cent).

All analyses (Figures S6–S9) yield broadly similar topologies, and we present in Figure 5 the results of the analysis with a binary covarion model, a relaxed clock, and a heterogeneous rate by part of speech. Despite the fact that our analysis only used two dating priors (the age of SWT and that of Ahom) instead of eight as in Tao et al. (2023), our results for the topology and the dating are very similar to those obtained in that study.

**Figure 6.**
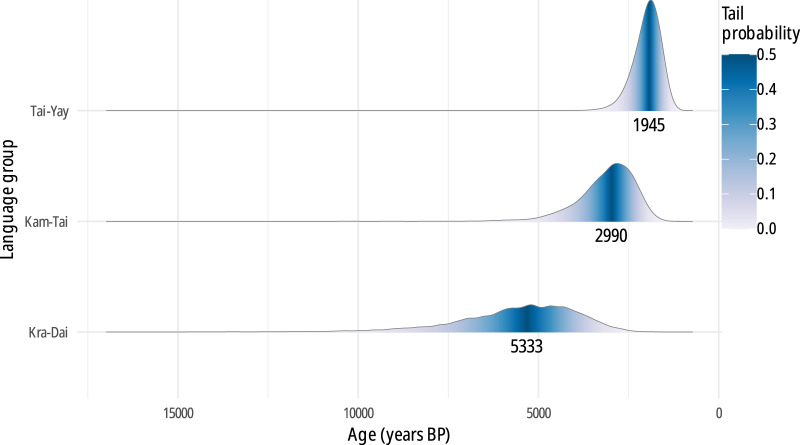
Probability density function and median values of the distribution of ages (years BP) for the most recent common ancestors (divergence times) of Kra-Dai, Kam-Tai, and Tai-Yay languages (binary covarion, relaxed clock, heterogeneous rates).

Figure 6 and Table S4 present the age distributions for the most recent common ancestors (MRCA) of Kra-Dai, Kam-Tai, and Tai-Yay in the relaxed clock with heterogeneous rate analysis. The strict clock analysis gives a considerably earlier and unrealistic root age (median age 8,966 instead of 5,333 with a relaxed clock), presumably due to the longer branch length of the Kra group, which is caused by a combination of factors. First, while the Kra languages have diverged early from the rest of Kra-Dai, many languages in this group have undergone a significant number of sound changes, and it is possible that their lexicons also evolved faster than that of the rest of Kra-Dai. Second, although Ostapirat (2000) elucidates the sound correspondences for seven Kra languages, we lack them for most Gelao languages, making it likely that cognates have been missed, thus biasing the age of the family. This issue can only be solved by further studies on the historical phonology of this subgroup, and goes beyond the scope of this work. In Tao et al. (2023), this problem was addressed by adding a temporal prior on the Kra node. However, there is currently little direct linguistic evidence supporting this prior.^2^ It is not surprising that a strict clock model with very few constrained ages yields unreliable age estimates.

Unlike some language families with rake-like tree topologies, such as Sino-Tibetan (Sagart et al., 2019), Kra-Dai presents a nested topology. All analyses have a Tai-Yay subgroup comprising Northern, Central, and South western Tai with high posterior probabilities (100% for the homogeneous rate analyses, 99% for the heterogeneous rate analysis). There is reasonably good support for a Be-Kam-Sui-Tai branch, within which Ong-Be is the outgroup, with a Kam-Tai clade comprising Kam-Sui and Tai-Yay (99% and 76% posterior probability in the strict clock and relaxed clock analyses, respectively). The same nesting has been proposed by historical linguists using traditional methods based on phonological and lexical innovations (Ostapirat, 2000, Norquest, 2015, Chen, 2018). Within Tai-Yay, there is little support in the relaxed clock phylogenies for the ‘Central Tai’ grouping that has been accepted in most previous work on Kra-Dai languages since Haudricourt (1956). The Longzhou, Debao, Cao Bang, and Wenma languages group with SWT, whereas others (Yongning, Long’an, Qinzhou, Shangsi, Fusui) appear closer to Northern Tai. In the strict clock phylogenies, by contrast, the only ‘Central Tai’ language to group with SWT is Wenma, while the other Central Tai languages appear as outgroups of the Northern Tai languages (except Saek, which is known to have highly archaic phonological features). With exception of these ‘Central Tai’ languages however, the results are broadly compatible with the traditional views on the phylogeny of Kra-Dai based on shared innovations (Ostapirat, 2000, Norquest, 2015).

### 3.6. The loom phylogeny

**Figure 7.**
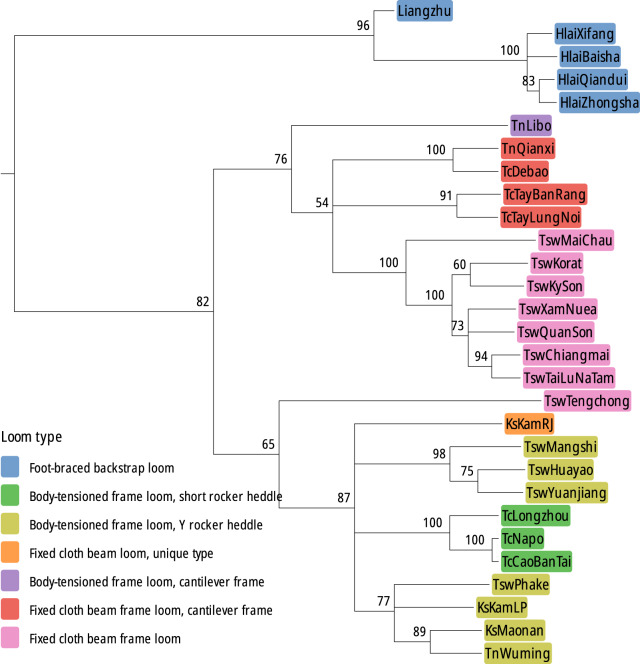
Majority-rule consensus tree for the looms, with all traits and no weighting (binary covarion, relaxed clock, heterogeneous rate); each node is annotated for its posterior probability (in per cent). The clades are split at a deep level between the frameless Hainan (Hlai) looms and looms with frames, which are further split between looms that have their cloth beams fixed to the weaver’s waist (body-tensioned) and those with fixed cloth beams. For detailed description, see the text (Section 4.1).

For the looms, the best performing model as measured by ML score was the binary covarion substitution model with a relaxed clock and a heterogeneous rate (see Supplementary Information), though we found that the inferred topology of the tree was robust as far as model choice is concerned. The consensus tree is shown in Figure 7. As with languages, looms present a well-resolved and deeply nested phylogeny. The simple foot-braced looms of the Hainan islanders group with the Liangzhu archaeological loom (which, aside from its fragmentary state, is essentially indistinguishable from the Hainan looms) and form an outgroup in relation to the looms with frames used by mainland Kra-Dai groups. The mainland looms are split at a deep level between body-tensioned types with a short rocker heddle and two groups of body-tensioned looms with long rocker heddles. A further clade contains a large group of looms with fixed cloth beams (i.e. not body tensioned) that are mainly used by groups speaking SWT languages, distributed from the China–Vietnam border region across Laos, Thailand, and Myanmar.

## 4. Discussion

**Figure 8.**
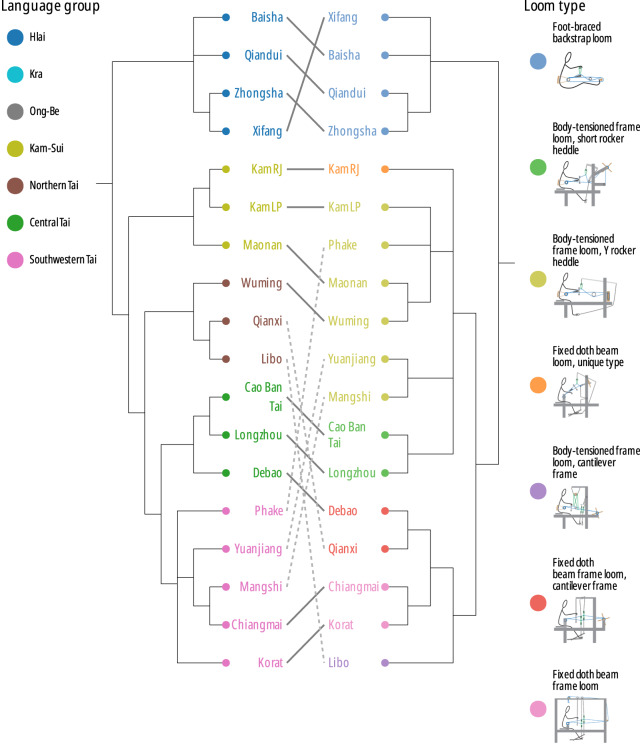
Comparison of majority-rule consensus trees computed for languages (left) and looms (right), restricted to tips present in both data sets. Despite many differences in detail, the two trees have broadly similar structures. The solid lines represent correspondences that are compatible with both phylogenies and the dashed lines those that present conflicting signal. The most divergent looms and languages versus the rest of the Kra-Dai peoples are those of the Hlai peoples (mid-blue, near the top of the diagram). The centre part of the trees are occupied by language speakers who stayed near to the homelands of Tai peoples in Guizhou and Guangxi provinces in China, who retain older loom forms with L-shaped frames. SWT speakers, with more innovative looms appear in the lower third of the figure, spread across the region from the China–Vietnam border into Laos and Thailand. Note that the trees are displayed as ultrametric, with the tips lined up in the centre of the plot for ease of readability, but the actual consensus trees are not ultrametric.

**Figure 9.**
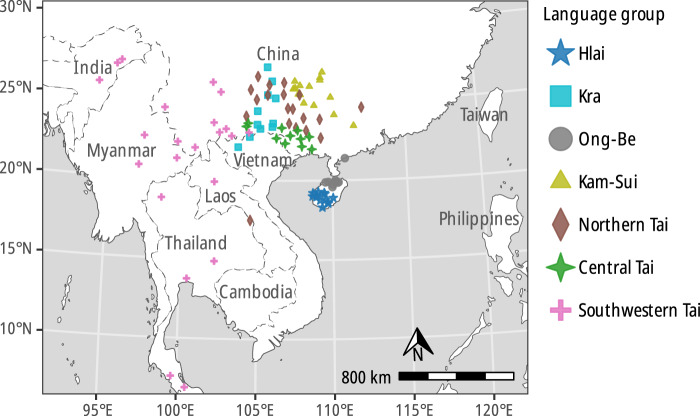
Locations of the Kra-Dai languages in the dataset, coloured according to the clades that they belong to; the labels ‘Northern Tai’, ‘Central Tai’ and ‘Southwestern Tai’ are assignments based on earlier work: The Central Tai grouping is not supported in this dataset.

**Figure 10.**
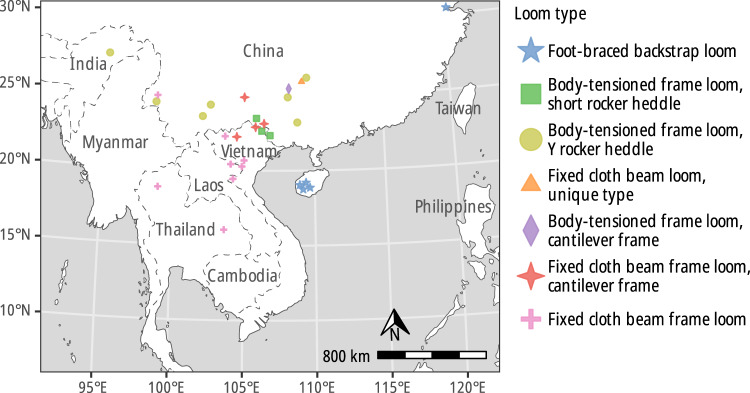
Locations of the Kra-Dai looms in the dataset, coloured according to the clades that they belong to.

We begin by reviewing the similarities and differences in the loom and language phylogenies and their implications for the historical migrations of the Kra-Dai peoples, before moving on to discuss more general topics related to the evolution of languages versus technologies. The consensus phylogenetic trees for both languages and looms are compared in Figure 8 (for the sake of clarity, only those taxa present in both data sets are shown). The locations of the languages and looms, colour-coded according to the clades in the consensus trees, are shown in Figures 9 and 10.

### 4.1. Commonalities

The language and loom phylogenies share a number of common features in their topologies. Both show an early split between the Hlai of Hainan and the rest of the Kra-Dai family, and their nested structures mirror the geographic locations of the taxa and presumed migration pathways. Pittayaporn (2014) showed that the final phase of Kra-Dai migration from the China–Vietnam border through Southeast Asia, and the corresponding diversification of SWT languages (and, by implication, the distinctive frame looms used by these peoples) took place within the last millennium.

Comparing the loom phylogeny in Figure 7 with Figure 3 shows that the different clades are mostly defined by structural features of the frames, and that these appear to have built up in a step-wise fashion, with newer features adding to rather than replacing earlier features. This property emerges naturally from the phylogenetic analysis and is independent of our assignment of levels to loom features. We can also see that looms with simpler frames (mainly L-shaped, with Level 1 and Level 2 features) are found near the presumed origin of Tai-speaking peoples in southern China, whereas looms with more complex frames with Level 3 features (overhead bars and box-like frames) are found mainly in northern Vietnam, Laos, and Thailand, amongst speakers of SWT languages.

The loom designs on the bottom row of Figure 3 with fixed cloth beams and paired, linked heddles operated by foot-treadles are a globally important type that spread throughout central Asia and Europe, becoming widespread in western Europe by the 11th–13th centuries (Ø ye, 2016), later brought to the Americas by European migrants. The place of origin of these looms is unknown, but the diversity of types within this dataset, which is much greater than found in other parts of the world, makes this region, and Kra-Dai speakers in particular, strong candidates for involvement in their origin and early development. The loom used by people in the Libo region (speaking a Northern Tai language) is particularly interesting in this regard, since it is body-tensioned but incorporates several innovations associated with fixed cloth beam looms, such as paired linked heddles, a cantilever frame, and a round warp beam with spokes. In the phylogenetic tree, this loom belongs to the same clade as the fixed cloth beam looms. It may represent an early experiment with these novel features.

### 4.2. Measuring the similarity between language trees and loom trees

We also assessed quantitatively the degree of similarity between the trees for looms and languages. Various methodologies have been used in the literature for similar questions. For instance, Learmouth et al. (2024) give a visual comparison of trees similar to our Figure 8, and then give individual measures of phylogenetic signal for each trait; Brown et al. (2014) and Passmore et al. (2024) compute distance matrices between leaves in several data sets, and then perform correlation tests between these matrices. We wish to answer two questions: First, are the looms and language phylogenies compatible? Second, is it reasonable to assume that looms and languages evolved along the exact same tree?

For the first question, we pruned the data and trees to keep only leaves that are present in both data set and performed two statistical tests. First, we compared the collection of distances represented by the consensus trees. For each pair of leaves, we computed the patristic distance on the consensus trees, in a similar fashion to Brown et al. (2014): This corresponds to the length of the path between two leaves, via their most recent common ancestor. This procedure gave a matrix of distances between languages and a matrix of distances between looms. We found significant evidence in favour of a correlation between the two matrices of distances (Mantel test *z* = 245.17, *p* < .001). This gives an initial quantitative measure of a feature that is apparent by inspection: That the two phylogenies are similar, although it does not take into account the posterior uncertainty in the reconstructed histories. We repeated this analysis by averaging over 1,000 trees sampled from the posterior on each side, normalising all branch lengths by the tree depth to avoid giving undue weight to deeper trees. For each analysis and each pair of leaves, we computed the mean distance across trees. We compared the two matrices of mean distances and again found significant evidence in favour of correlation (Mantel test *z* = 292.94, *p* < .001).

Second, we measured whether the loom tree provides good explanatory power for the language data and vice versa. To this end, we performed a principal component analysis on the binary data at the leaves. We then computed Blomberg’s *K* (Blomberg et al., 2003), which measures the amount of phylogenetic signal in data at the tips of the tree, mapping the first component of the looms data onto the language trees (binary covarion, relaxed clock, heterogeneous rates) and that of the linguistic data onto the looms trees (binary covarion, relaxed clock, heterogeneous rates, all levels, no weighting), averaging in each case over all trees in the posterior sample. Mapping the looms data onto the posterior language trees, we find *K* = 5.87 (*p* = .001); mapping the language data onto the posterior looms trees, we find *K* = 2.22 (*p* = .001). Blomberg’s *K* computes the ratio of the observed variance explained by the phylogeny to the variance expected under a random model; values greater than 1, as seen with *K* = 5.87 and *K* = 2.22 indicate a strong phylogenetic signal. *K* = 5.87 suggests the language phylogeny explains a significant part of the variation in looms data.

Note that we have not explored the potential impact of geography on these correlations but leave these considerations to future work.

For the second question, we compared different models using Bayes factors. We computed the marginal likelihood for two scenarios: (A) looms and languages evolved along the same phylogeny, and (B) looms and languages evolved along two different phylogenies. For that, we restricted our analysis to those taxa present in both data sets, and we used the same model on both datasets (binary covarion, strict clock, homogeneous rate). The pruned data contains no fossils, so we used a birth–death model tree prior, and we constrained the age of the MRCA of the Li languages (Meifu, Ha, Qi, and Run) to be between 500 and 1,500 years BP. Scenario A means both looms and language traits evolved along the same single phylogenetic tree. We thus merged the looms and language character matrices into one and ran the analysis. On the other hand, scenario B assumes looms and language traits evolved along different phylogenies. We thus analysed separately the two datasets and summed the marginal likelihoods of the two analyses. We found a marginal log likelihood of 
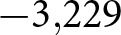
 (with standard deviation of 5.02) for scenario A, and of 
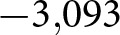
 (with standard deviation of 4.18) for scenario B. This corresponds to a log-Bayes factor of 135 in favour of model B. Following the standard scale of Kass and Raftery (1995), there is thus decisive evidence in favour of scenario B: we conclude that despite the similarities discussed above, the language and loom phylogenies are distinct.

### 4.3. Differences

Aside from the common features discussed above, there are also some important differences between loom and language phylogenies, some of which have implications for the early history and migrations of the Kra-Dai peoples.

#### 4.3.1. Tai Phake in Assam

The Tai Phake people in Assam speak a SWT language but use an ancient body-tensioned loom with an L-shaped frame (a combination of Level 1 and Level 2 frame features), which belongs to the same clade as the Kam loom (looms 2 and 3 in Figure 3) and is closely related to looms used by Tai-speaking peoples in Yunnan. It is quite different from SWT looms and Indian-influenced looms used by their neighbours: the Aiton and Ahom. They are unlikely to have acquired this during their migration to their present location, since neighbouring peoples do not use such looms. The possibility that the Tai Phake once had a more sophisticated loom but subsequently reverted to this older type seems unlikely.

Two hypotheses can be proposed to account for the archaism of the Tai Phake loom. First, Tai Phake could have begun to move away from other SWT language speakers before the emergence of more sophisticated looms, thus retaining an older type. Second, the Tai Phake could instead have been an unrelated group who shifted their language to SWT during the last millennium but kept their original loom. At present, there is little historical evidence to decide between these two hypotheses. Morey (2004, 208) reports that ‘[t]he traditional view is that the Aiton, Khamti, Khamyang, Phake and Turung all entered Assam between the middle of the eighteenth century and the early nineteenth century, having migrated from Burma and bringing with them Theravada Buddhist religion and scripts which are closely related to the Shan of Burma’, but at the same time he notes that oral traditions among the Aiton and other Tai groups of Assam claim that their migrations date from the arrival of Prince Sukhapha in the 13th century.

#### 4.3.2. Dai in Yunnan

A second difference concerns the looms used by some Dai (SWT) speakers in Yunnan in the Yuanjiang (元江) and Mangshi (芒市) regions, and the ‘Huayao Dai’ in the Yuxi (玉溪) region. As with the Tai Phake, despite speaking SWT languages these groups retain an older type of loom with an L-shaped frame that split off from other loom designs at a deeper and earlier date than is implied by their linguistic affiliations. The Yuanjiang and Mangshi weavers also possess complex pattern heddles, however, that are closely related to systems used by SWT-speaking weavers in northern Vietnam. As with the Tai Phake, we suggest that these groups have retained a very early loom technology, with more recent addition of a patterning system and (probably) language change as a result of contact with SWT speakers in southern Yunnan.

Archaeological evidence suggests that the loom types used by the Tai Phake and the Yunnan Dai weavers developed more than 2,500 years ago (Buckley, 2023). Loom types with fixed cloth beams are a more recent development. The close association between fixed cloth beam looms (of several different forms) and SWT speakers suggests that the differentiation of these looms occurred in parallel with that of the language groups, presumably during the last 1,000 years.

**Figure 11.**
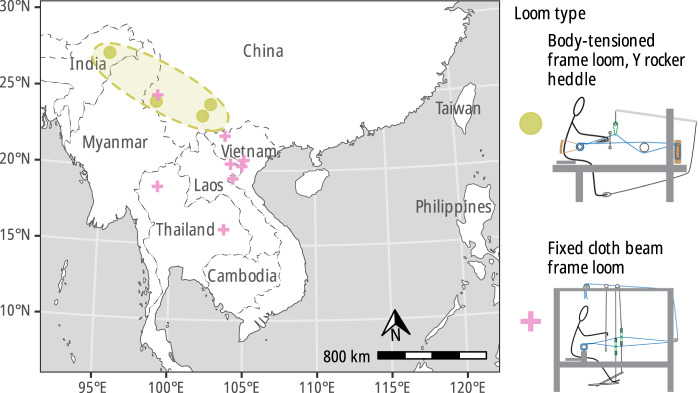
Locations of SWT-speaking peoples in Yunnan and Assam using archaic loom technologies with L-shaped frames (green dots); these technologies are not closely related to the fixed cloth beam frame looms used by other SWT speakers (pink crosses) but are closer to ancestral looms still found in the Tai homeland region in Guizhou/Guangxi provinces in China.

As in the case of the Tai Phake, the retention of ‘fossil’ looms among the Yunnan Dai people (Figure 11) could be accounted for by two main hypotheses: either language shift of earlier non-SWT populations or preservation of the original loom types used by Proto-SWT people before the spread of fixed cloth beam frame looms. In the former case, this would imply that some SWT-speaking groups either were originally non-Kra-Dai-speaking (in particular Austroasiatic) or were a non-SWT-speaking Kra-Dai people who migrated to Yunnan and further south earlier than SWT, later shifting to an SWT language.

#### 4.3.3. Zhuang and Nung speakers in the China–Vietnam border region

A further puzzle concerns the body-tensioned frame looms with short rocker-heddles (another older type based upon Level 1 and Level 2 frame features, corresponding to looms 4 and 5 in Figure 3) used by small groups of Central Tai speakers in the border region between north Vietnam and China, speaking languages usually referred to as ‘Zhuang’ (壮族) in China and ‘Nung’ in Vietnam. This loom is rare in southwest China but is found amongst rural Han Chinese speakers across a wide region of central China. Independent invention of this complex loom by Kra-Dai speakers and Sinitic speakers is unlikely, which implies that horizontal transfer of either loom or language has occurred. There seem to be two main possibilities: one is that Kra-Dai speakers were in contact with Sinitic speakers at an early date and acquired the loom from them. The other is that these groups were originally Sinitic or other language speakers who underwent language shift to Central Tai during the last millennium, retaining their original looms. We note, however, that the word for ‘loom’ in these languages (e.g. Longzhou *huk^7^*) reflects the Proto-Tai etymon *truk^D^ (Pittayaporn, 2009, 147) and is not borrowed from Sinitic.

### 4.4. Modular organisation and the question of ‘design’

Our results show that looms are composed of technological modules in which groupings of features are built upon Level 1 and Level 2 choices, particularly in frame components and that these are defining features of loom clades. There is no evidence in our study, however, that this modularity is the result of strategic planning on the part of loom builders. On the contrary, it seems to have arisen as a result of incremental trial and error processes, coupled with evolutionary constraint for features that accomplish the weaving task successfully (but not necessarily optimally). The evidence for this is the very wide variety of loom types and modules found amongst Kra-Dai weavers (and other linguistic groups in the same region), as illustrated in Figure 3. This is the opposite of the pattern that would be expected if weavers had set out to design optimal looms based on a general concept of weaving, or to copy their neighbour’s designs with the idea of selecting the best available method. Instead, weavers have followed forking paths to arrive at diverse solutions for the same basic problem (interlacing warp and weft). Most of these incremental developments seem to have occurred locally and independently, though there is evidence (discussed below) for the replacement of older body-tensioned designs by fixed cloth beam looms (a more efficient system) by some groups.

As one of our reviewers pointed out, the designs of looms are path dependent: the loom that you end up with depends very much on the one that your ancestors began with. This is an indication that complex looms with frames, despite their diversity, only explore a part of the available design space (morphospace). Despite similar needs, neighbouring groups have not converged on identical or even similar loom designs.

Once established, features at Level 1 and 2 become the basis for suites of associated features (modules). These features cannot be easily changed without a radical change to the loom design. Thus, the evolutionary rates of change for these features are slower than for features at higher levels.

### Mode and tempo in weaving technology and language evolution

Comparison of the evolution of languages and looms in the two datasets reveals differences in the ways in which linguistic features and technologies evolve. Comparing across groups, the Kra-Dai languages appear to have evolved in (broadly speaking) similar ways and at similar rates, permitting estimates of divergence times. In contrast, loom technologies show several distinct modes that are (considered as a whole) characteristic of *punctuated evolution*:

- **Stasis**: as noted, looms used by the Hlai-speaking peoples of Hainan share a clade with an archaeological loom from the Liangzhu culture. These looms have undergone only minor changes over four millennia or more.
- **Progressive change at varying rates**: looms and patterning systems used by SWT speakers in northern Vietnam and Laos show a progression of forms at a faster tempo, with the appearance of new types of frames based around Level 3 features. Most of these developments occurred during the last millennium. At the same time, older looms based upon Level 1 and Level 2 features (only) persisted near the homeland of Tai speakers in southern China.
- **Abrupt change due to horizontal transfer**: for example, looms used by Tai speakers in Qianxi (黔西), Debao (德保), and Tengchong (腾冲) appear to have acquired fixed cloth beams by horizontal transfer, since there is no evidence of the gradual development of this feature within their lineages. The Qianxi and Debao looms with fixed cloth beams and cantilever frames probably represent complete replacements of earlier loom types. The Tengchong loom is a hybrid, which probably accounts for its inconsistent placing within the loom phylogenies described above.

These differences seem to be due to fundamental differences between languages and physical technologies. In the former, cognate words can vary virtually independently of each other. The degree of innovation required to alter a particular word is minimal, and random changes are generally harmless, ie: they have a negligible impact on cultural or biological fitness. We would therefore expect cognate words to evolve mainly by a process of drift, since few changes can be said to offer clear advantages to their users, except perhaps in differentiating them from their neighbours.

In contrast, in the case of looms, many characters are interdependent to a greater or lesser degree. Loom features are also strongly linked to external constraints: random changes, for example, are likely to damage the functionality of the loom. Innovative thinking is required in order to make useful changes, though if successful, these may offer real advantages to their users, for example, in efficiency of weaving, or in recording patterns for future use.

Complex innovations come at a price of more investment of materials and labour in loom construction and in weaver’s time and effort in learning how to use them. On the one hand, Hlai weavers in Hainan have contented themselves for four millennia or more with the exploration of a limited part of the morphospace of loom forms, consisting of a few sticks that are linked together by the warp only when the loom is in use. On the other hand, mainland Tai weavers developed looms that placed the warp beam in a frame (and the weaver in a seat on that frame), a major step that opened up possibilities for the addition of new functional modules, as described above. This factor, associated with socio-economic changes, such as increased agricultural surplus amongst mainland wet-rice farmers, and endogenous factors, such as competition for social status and prestige, presumably created suitable conditions for a change in tempo from gradual to rapid accumulation of innovations. This is particularly apparent in the looms of relatively prosperous Tai speakers in northern Vietnam and Laos, associated with the production of fine silk textiles for ceremonial purposes and for trade, such as that shown in Figure 1.

Our analysis of the rates of changes of interdependent features at different levels reveals a further interesting aspect of technological evolution. There is significant variation in the rates of evolution of various features (Figure 4), with fundamental, independent traits (Level 1) evolving at a slower pace than dependent traits (Levels 2 and 3). Broadly, this provides further support for the view that technological evolution is modular, the modules being defined by Level 1 and Level 2 traits and their dependent features. Modules, once established, are relatively stable: they are the main defining feature of clades in the phylogenetic tree of looms. Evolution within modules can proceed at a more rapid pace, however, producing a diversity of forms. To our knowledge, this is the first time this kind of systematic level-based analysis has been employed to investigate technological evolution.

We also note a basic difference between our looms and language datasets. In the case of looms, our data includes all (or nearly all) of the functional traits of these technologies. Many of these traits are present in some looms but not in others: The number of positive traits varies between 22 for the simple Liangzhu loom and 60–80 for the complex frame looms. In the case of languages, the dataset consists of a carefully chosen list of around 90 concepts, focusing on universals such as ‘water’, ‘tree’, ‘nose’, ‘leaf’, etc. that are are expected to be present in all (or nearly all), languages, and are presumed to be revealing of deep roots and relationships between languages. Had we chosen instead to examine a different subset of language, such as terms of more recent origin, the dynamics that we uncovered would undoubtedly have been different.

### Gradual vs punctuated change

Darwin originally conceived of evolution as a gradual process. In contrast, Eldredge and Gould (1972) noticed that much evolutionary change is in fact characterised by periods of stasis interrupted by short bursts of change, which they termed ‘punctuated equilibria’. Their observation turned out to be a general phenomenon, found across a range of evolutionary processes, both biological and cultural. It has strong theoretical underpinnings, associated with the behaviour of linked hierarchical evolving units (modules), particularly in the presence of feedback loops, as discussed by Duran-Nebreda et al. (2024). Punctuated change seems to be a characteristic feature of human technological evolution, observed in fields as varied as looms (as described here), programming languages (Valverde and Solé, 2015), and the sizes of seagoing vessels (Pascali, 2017, AIimpactswiki, 2024). It is also apparent in the morphology of organisms, which undergo adaptive radiations interspersed with periods of relative stasis.

In contrast, gradual change is prominent in two areas: molecular DNA sequences and word-lists for basic concepts such as the one we have used. Both of these can be expected to evolve mainly by a process of neutral drift, i.e. the accumulation of random changes. The neutral model of genetic evolution and its application to molecular sequences have been widely used and discussed, but the application of neutral models to language change has received more limited theoretical attention to date (but see Reali and Griffiths, 2010). Nevertheless, the use of clock models in linguistics, as well as biology, to estimate divergence times rests on an implied theoretical foundation of neutral evolution, ultimately derived from the work of Kimura (1968).

Why do some evolving systems (and datasets) display gradual change, while others evolve in bursts? There is a clue in the comparison of the evolution of protein molecular sequences and structures, investigated by Pascual-García et al. (2019). The authors found that violations of strict clock-like behaviour were found in both sequences and structures but were greater in structures, and they suggested that the latter are more strongly linked to, and constrained by, selection pressures than the former.

**Figure 12.**
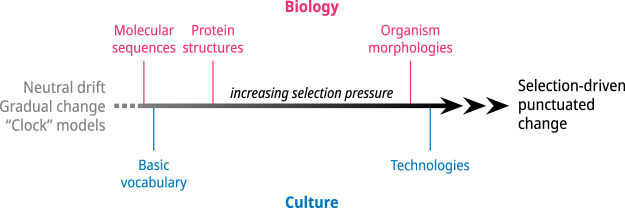
Conceptual sketch of the link between empirically observed modes of evolution (gradual versus punctuated change) and selection pressure (the horizontal scale is arbitrary and is not intended to depict precise relationships).

Despite the apparent gulf between protein biology on the one hand and Kra-Dai cultural features on the other, we suggest that similar contrasts in evolutionary dynamics may be operating in both cases. The key to this is recognising that evolutionary processes occupy positions on a sliding scale: at one end of the scale, there are those that are weakly linked to external selection pressures and are thus mainly subject to neutral drift, such as nucleotide base pairs and basic vocabulary. At the other end lie processes that are strongly linked to external forces (selection pressures and features of the morphospace), such as the evolution of complex looms with frames. The middle ground is occupied by features such as protein structures that are moderately coupled to external factors and also subject to neutral drift (Figure 12).

There is evidence that even languages may evolve in bursts on occasions, as shown by Atkinson et al. (2008). Their results suggest that ‘pure’ gradual evolutionary change may not exist in nature, even though change in language core vocabulary approaches this condition sufficiently closely for clock models to yield useful results (Greenhill et al., 2020). Atkinson et al. (2008) make the important point that ‘bursts’ in language evolution are not intrinsic to language change but are linked to transitory external pressures, such as language contact.

In the case of looms, the punctuational nature of their evolution is intrinsic and linked to the nature of the morphospace. The appearance of new features makes new regions of the morphospace accessible and permits bursts of evolution as these regions are explored. There are also links with external factors, such as economic productivity and commercial and social pressures, that may also promote bursts of evolutionary change. They may also prevent them, for example, constraining resource-poor groups such as the Hainan islanders to use the same simple loom design for an extended period.

These differences highlight the uniqueness of language amongst aspects of human culture and suggest reasons why we should not expect language to evolve in tandem with material culture. Generalising from our observations and those of other authors, we suggest that the ‘smoothness’ of evolution of both cultural and biological features is determined in large part by the degree to which they are subject to external selection pressures.

## 5. Conclusions

We have directly compared the evolution of two aspects of culture, language and weaving technology, within a single group of peoples, the Kra-Dai, who are dispersed across southern China and mainland Southeast Asia. The comparison yields insights into the history and migrations of the Kra-Dai, as well as the nature of evolutionary change.

Looms and language evolve along related but different paths. In the case of the Kra-Dai, this confirms the overall picture of early migration from the Yangtze River region followed by diversification near what is now the China–Vietnam border, followed by a major expansion of SWT speakers into Southeast Asia during the last millennium. It also hints at earlier migrations (occurring before the SWT expansion) to Yunnan and the China–Myanmar border region that may have left technological ‘fossils’ in their wake. The view obtained from looms and languages is thus a complementary one. We have broken down the evolution of loom technology by level, showing that the deeper, more fundamental features that define the modular architecture of looms evolve at a slower pace than features at higher levels.

To answer the question set out at the beginning of this paper, despite similarities in the ways in which languages and material cultures are transmitted, we can expect their modes of evolution to be different – the former evolving at a relatively steady pace, and the latter evolving in bursts (punctuated evolution). By comparison with results from widely divergent fields in biology and culture, we suggest that these differences are examples of a general phenomenon, linked to the intensity of selection pressure experienced by the target of evolution.

## Supporting information

Buckley et al. supplementary material Buckley et al. supplementary material

## Footnotes

1 Kra-Dai languages have no conjugation or declension, and therefore word classes are identified on the basis of word order or distributional properties.

2 Chinese loanwords could provide evidence for dating Proto-Kra or subgroups thereof, but none have been discovered up to now.

## Supplementary material

The supplementary material for this article can be found at https://doi.org/10.1017/ehs.2025.10008.

## Research transparency and reproducibility

All the data and computer code used for this study are available at https://doi.org/10.17605/OSF.IO/6V9RS.
