# Supplementary material for: Contrasting modes of cultural evolution: Kra-Dai languages and weaving technologies
Source: Evol Hum Sci. 2025 Jul 25;7:e35. doi: 10.1017/ehs.2025.10008 (PMC12645327; doi:10.1017/ehs.2025.10008)
Supplement: Buckley et al. supplementary material [file S2513843X2510008Xsup001.pdf]

February 24, 2025

SUPPORTING INFORMATION

Contrasting modes of cultural evolution: Kra-Dai languages and weaving technologies

Christopher D. Buckley,[1] Emma Kopp,[2] Thomas Pellard,[3] Robin J. Ryder,[4] and Guillaume Jacques[*,3,5]

[1]Tracing Patterns Foundation, Berkeley, CA
[2]CEREMADE, CNRS, Université Paris-Dauphine, PSL University
[3]CRLAO (EHESS, CNRS, Inalco)
[4]Department of Mathematics, Imperial College London
[5]École Pratique des Hautes Études, PSL University
*Corresponding author. Email: rgyalrongskad@gmail.com

1. Data and methods

The linguistic and looms data were prepared in the form of binary character state matrices in NEXUS format with the help of the R software (R Core Team 2024, version 4.4.1) and the packages `tidyverse` (Wickham et al. 2019), `phangorn` (Schliep et al. 2016) and `TreeTools` (Smith 2019). For the weighted analysis, we prepared a NEXUS file with some traits duplicated, i.e. Level 1 trait columns appear 8 times each, Level 2 trait ones 4 times, Level 3 traits ones twice, and Level 4 ones only once.

These were then analyzed in BEAST 2.6.7 (Bouckaert et al. 2019) with a coupled MCMC algorithm. For each analysis, the chain length was set to 100 million iterations and the sampling frequency to 10 000. Convergence was confirmed in Tracer 1.7.2 (Rambaut et al. 2018) by visually inspecting the resulting traces and checking that all effective sample sizes were greater than 200, following the removal of 10% burn-in.

For looms, we carried out analyses using a fossilized birth-death model tree prior (Gavryushkina et al. 2014), with a uniform prior [4.3, 5.3] on the age of the Liangzhu tip (following Qin 2013). Most analyses used a strict clock model and a binary covarion model of trait evolution that allows traits to switch between hidden states with slow and fast rates of change (Tuffley & Steel 1998, Penny et al. 2001). We carried out the following analyses:

1. Level 1 traits only;
2. all traits and no weighting;
3. all traits weighted by level;
4. basic traits only;
5. patterning traits only.

Each of these analyses was run with a single rate of mutation and then with traits partitioned by level and one mutation rate per level (except for the analysis using Level 1 traits only).

We also included the following analyses on all traits without weighting:

1. binary covarion model of trait evolution, relaxed log normal clock model (Drummond et al. 2006) (instead of a strict one), and a single mutation rate;
2. binary continuous-time Markov chain (CTMC) model of trait evolution (Gray & Atkinson 2003, Bouckaert et al. 2012) (instead of the binary covarion), strict clock model, and a single mutation rate;
3. binary CTMC model of trait evolution, strict clock model, and one mutation rate per level.

For languages, we used a binary covarion model and a fossilized birth-death model tree prior. We defined a uniform prior [0.3, 0.6] on the age of Ahom (following Morey 2004) and a uniform prior [1, 1.2] on the age of the most recent common ancestor of the Southwestern Tai languages (following Pittayaporn 2014), without assuming that they form a monophyletic group. We used a relaxed log normal clock model with either a single mutation rate or one rate per part of speech category (adjectives, nouns, verbs, others). We also included an analysis with a strict clock and a single mutation rate. For all analyses, we left all other settings at their default values in BEAST.

Majority-rule consensus topologies, mean edge lengths, ancestor ages, and highest posterior density intervals for mutation rates and ages were computed with the R packages `phangorn` (Schliep et al. 2016), `phytools` (Revell 2024), `HDIInterval` (Meredith & Kruschke 2022), `tracer` (Bilderbeek & Etienne 2018), and `tidyverse` (Wickham et al. 2019). Trees were annotated and visualized with the R package `ggtree` (Yu et al. 2017). Maps were produced using data from the public domain dataset of Natural Earth (`https://www.naturalearthdata.com`) with the R package `sf` (Pebesma 2018).

For studying the coevolution of looms and languages, we also prepared character state matrices for looms and languages restricted to common taxa, i.e. language groups for which looms data were available and vice versa, and a matrix merging both language and looms characters for common taxa. We then analysed these with a binary covarion model, a strict clock model, and a single mutation rate. Since the common taxa contain no fossils, we used a birth-death model tree prior.

We then run a principal component analysis (PCA) on the pruned language matrix and the pruned loom matrix with the R package `FactoMineR` (Lê, Josse & Husson 2008), and we extracted the first component for each. We then used the function `phylosig` in the R package `phytools` (Revell 2024) to compute Blomberg's K (Blomberg, Garland & Ives 2003), which measures the amount of phylogenetic signal in the data. We computed the value of K for the first component of the looms data mapped onto the posterior language trees (binary covarion, relaxed clock, heterogeneous rate), and for the first component of the linguistic data mapped onto the posterior looms trees (binary covarion, relaxed clock, heterogeneous rate, all levels, no weighting). In each case, we took the average values of K and its p-value over all trees in the posterior sample.

All the data and code used for the analyses are available at `https://doi.org/10.17605/OSF.IO/6V9RS`.

2. Results

2.1 Model selection

Table S1 presents the marginal likelihood for each model for the loom data. To ensure a fair comparison, we only considered models with identical weights. The calculation of marginal likelihood is based on the nested sampling algorithm (Maturana Russel et al. 2019), which is both computationally expensive and complex. Indeed, the algorithm often struggles to find an initialisation state. To facilitate the initialisation, we reparametrised the models to avoid improper priors and adjusted the Liangzhu calibration by applying a uniform prior in the range $[1.3, 50.3]$.

We found that the marginal likelihood calculations for more complex models were numerically unstable. In hindsight, this is not surprising: we are only constraining the age of a single internal node, and the parameters linked to the mutation rate may not be identifiable. The inference of multiple rates, such as mutation and clock rates, requires careful prior calibration because their proportional relationship means changes in one can be offset by the other, impacting the identifiability of branch lengths.

The strict clock model allows just one rate and represents a drastic simplification. On the other hand, the relaxed clock model allows rate variation between branches: to be able to reliably estimate the amount of variation (i.e. the variance of the distribution of rates), we would need multiple calibration points. This does not have a strong impact on the MCMC output of the relaxed clock model, but it does mean that the marginal likelihood is both numerically less stable and more sensitive to the prior

Table S1. Summary of the marginal log likelihood (ML) for different models for looms data.

	substitution	clock	rate	ML	sd
☞	binary covarion	relaxed	heterogeneous	-2025	6.62
	binary covarion	strict	heterogeneous	-2042	6.25
	CTMC	strict	heterogeneous	-2050	6.48
	binary covarion	strict	uniform	-2073	6.04
	binary covarion	relaxed	uniform	-2084	6.91
	CTMC	strict	uniform	-4213	0.02

choices. In practice, this makes it difficult to compute the Bayes factor to compare the relaxed and the strict clock models.

Since the parameter of interest is topology, and the inferred topologies are in agreement between the relaxed clock and the strict clock analyses, this problem is of little import. We do not make claims for the accuracy of the absolute ages estimated in our analyses. Our analyses do however yield useful insights into the differences in relative rates between loom features at different Levels.

2.2 Phylogenies

The majority-rule consensus trees for the different analyses of looms appear in Figures S1–S5. Figure S1 shows the results obtained on Level 1 traits only, i.e. the most fundamental defining features of the looms that are independent of one another. This tree reproduces the main clades within the full data analysis, but does not resolve the fine details of the topology. The trees in Figures S2 (all traits, unweighted, homogeneous rate) and S3 (all traits, weighted, homogeneous rate) show only minor differences in topology, from which we conclude that the analysis is not particularly sensitive to the type of weighting we use nor to the varying mutation rates.

Figure S5 shows the tree obtained by analysing the patterning systems (only). These are portable and easily transferred between looms: for example when a weaver marries into a neighbouring group she may leave her natal loom behind but take her patterns with her. The results confirm that patterning traits display an overall weak phylogenetic signal in comparison with looms.

Amongst all of these trees, the most important variation concerns the position of the Dai Tengchong loom. This loom is a hybrid formed from a Yunnan Dai body-tensioned loom with a fixed cloth beam grafted onto it (by horizontal transfer), and a patterning system that has probably been copied from South Western Tai looms. It thus has a conflicting set of features and does not group consistently with either the fixed cloth beam looms or the body-tensioned frame looms.

Figures S6–S9 show the majority-rule consensus trees for the linguistic data, with colors indicating the traditional subgroupings of Kra-Dai languages. The existence of most of these classical subgroupings is corroborated, except for the "Central Tai" grouping which is split between a northern and a southwestern clade.

2.3 Mutation rates and ages

Table S2 summarises the distribution of mutation rates of loom traits according to their level (binary covarion, relaxed clock, heterogeneous rate, all traits, no weighting).

Table S3 and Figure S10 present the mutation rates of language traits by part of speech (binary covarion, relaxed clock, heterogeneous rate), illustrating that nouns stand out among other word classes in having a slightly lower mutation rate.

Table S4 summarizes the posterior sample distribution of the ages of the root and two clades within the linguistic phylogeny (binary covarion relaxed clock, heterogeneous rate).

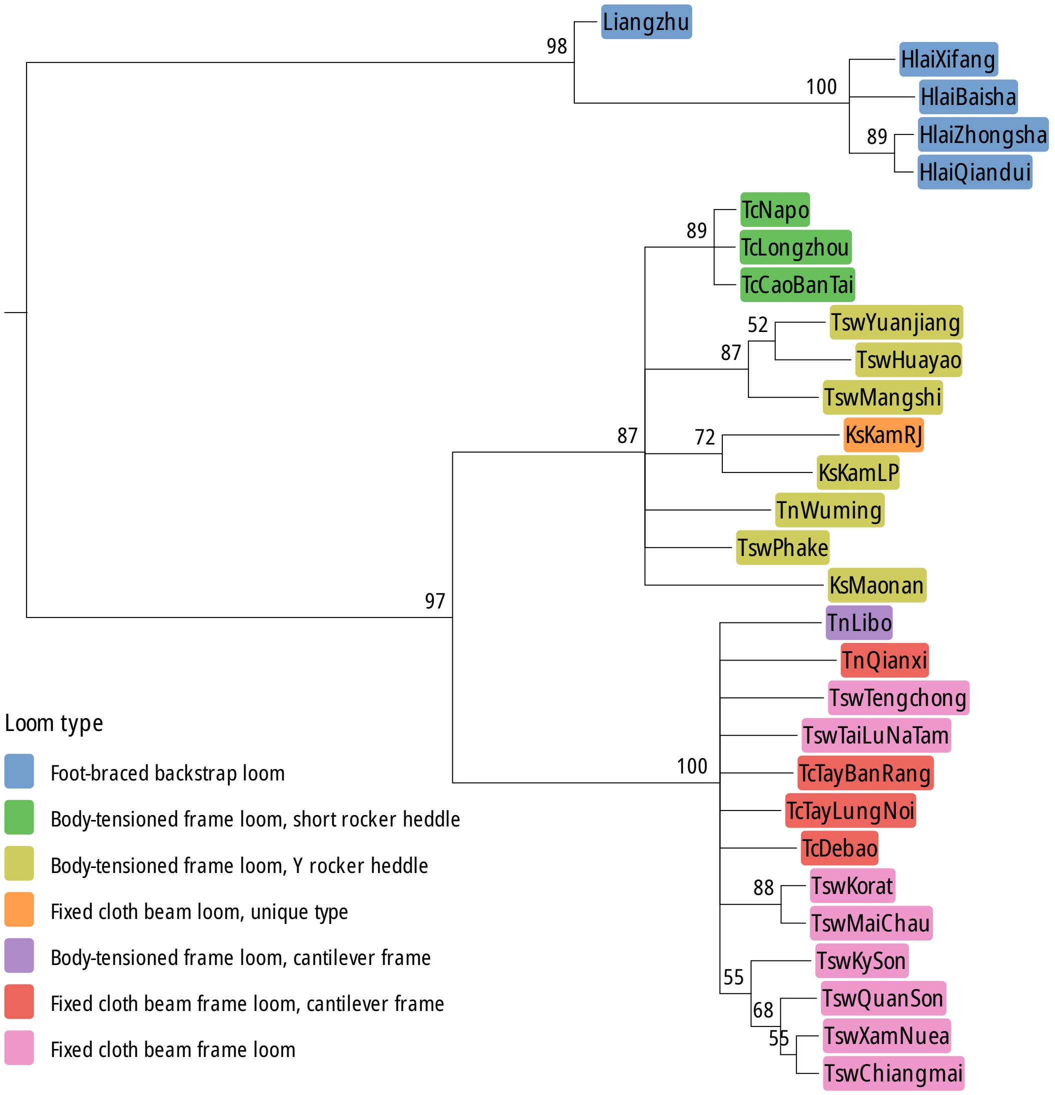

Figure S1. Majority-rule consensus tree for the looms, based on Level 1 traits only (binary covarion, strict clock, homogeneous rate); each node is annotated for its posterior probability (in percent).

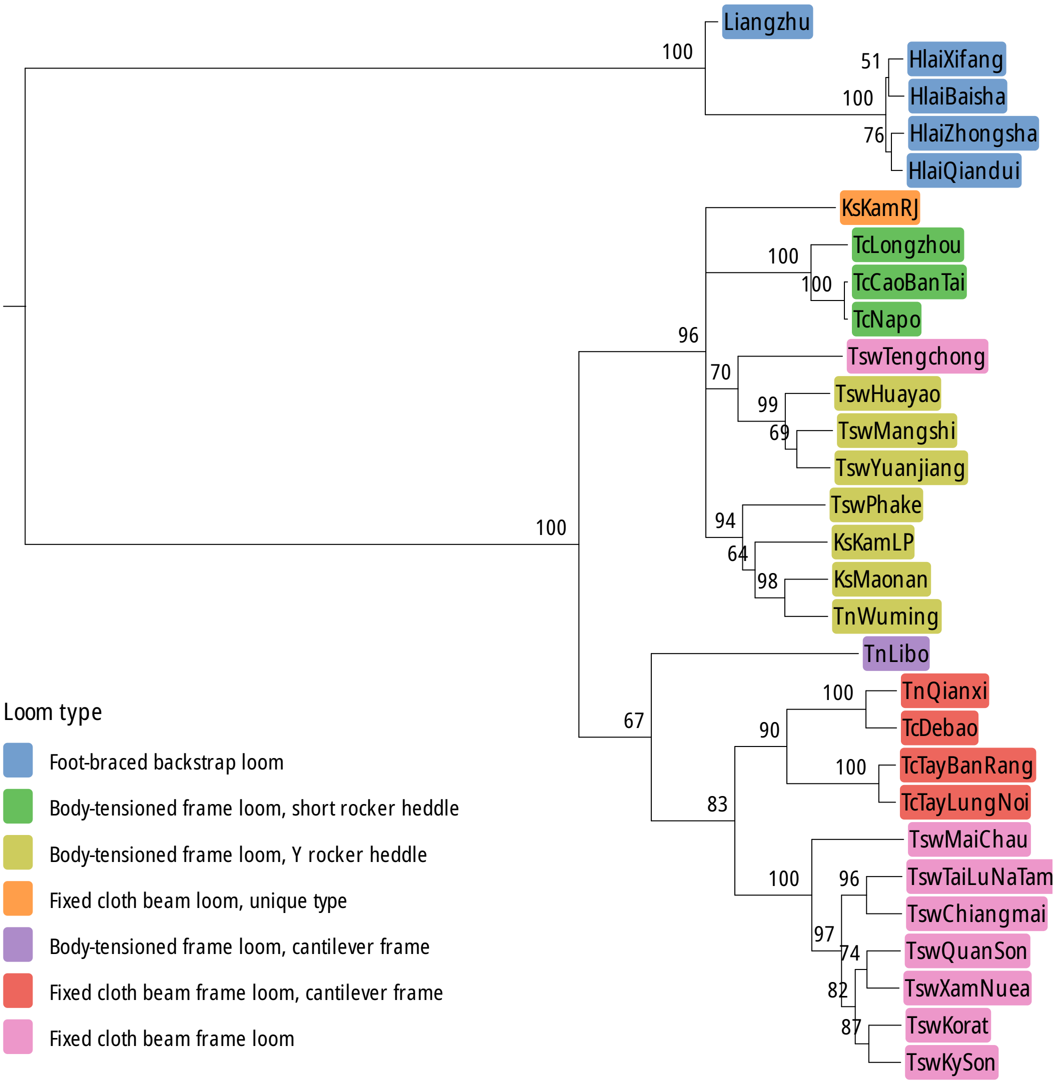

Figure S2. Majority-rule consensus tree for the looms (binary covarion, strict clock, homogeneous rate), without character weighting; each node is annotated for its posterior probability (in percent).

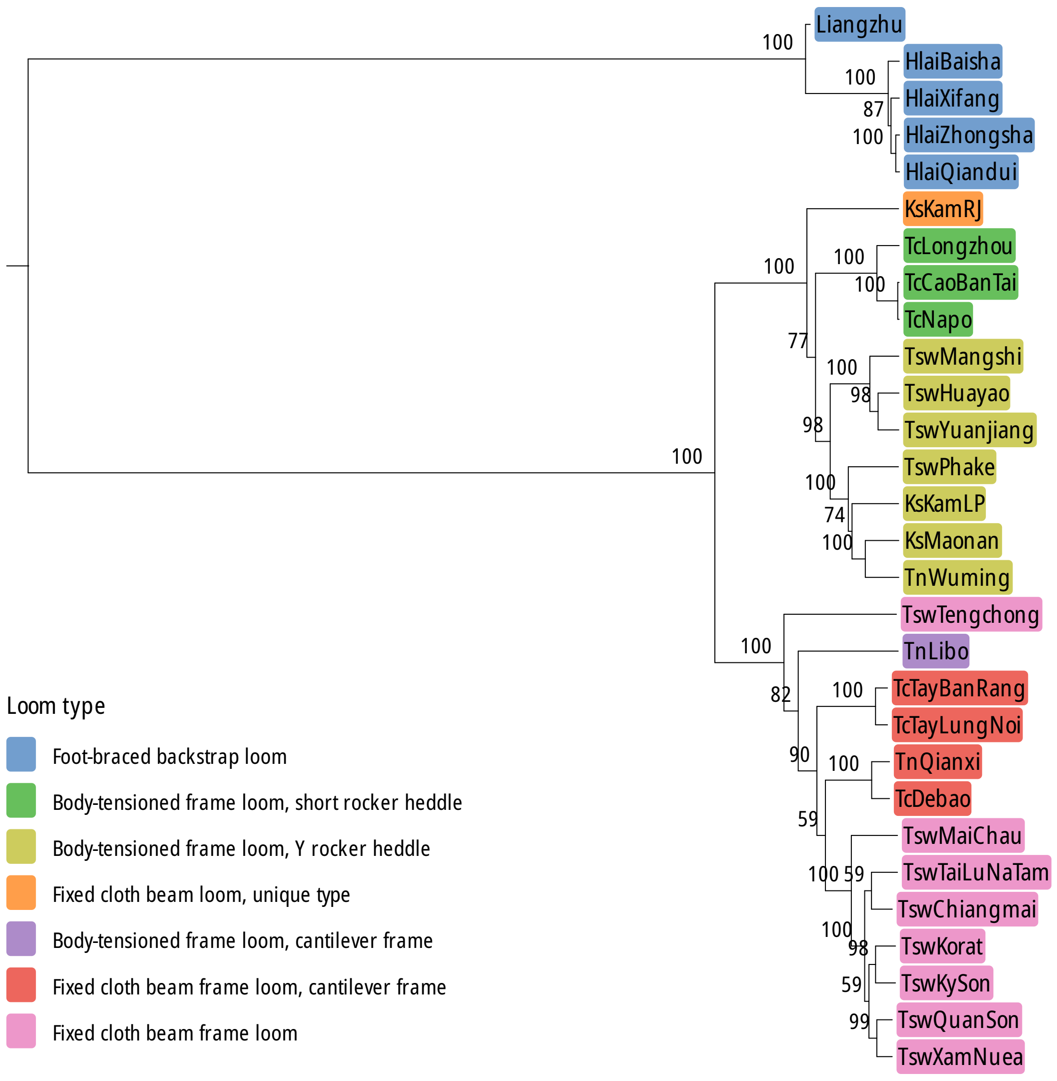

Figure S3. Majority-rule consensus tree for the looms, with different weights applied to levels (binary covarion, strict clock, homogeneous rate); each node is annotated for its posterior probability (in percent).

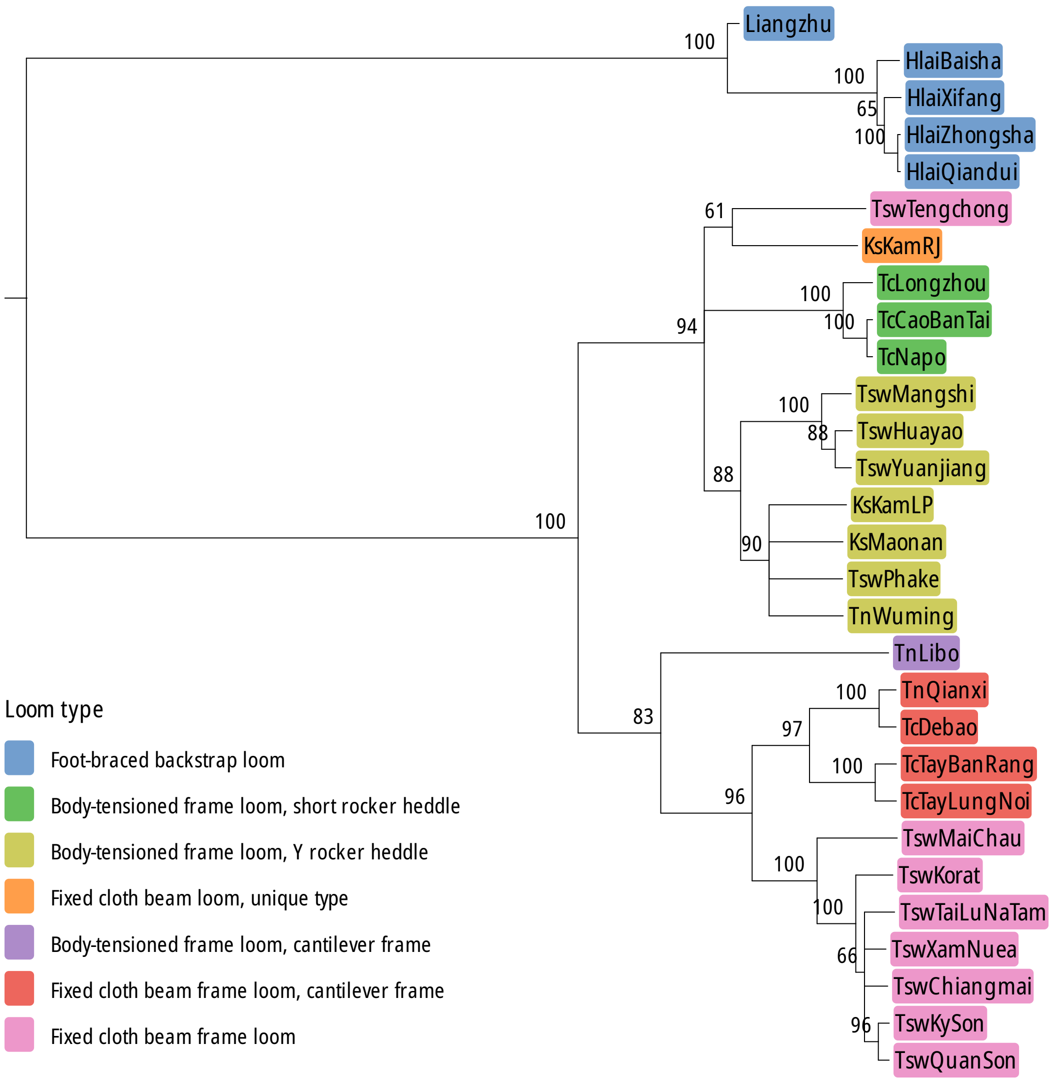

Figure S4. Majority-rule consensus tree for the looms, based on basic characters only (binary covarion, strict clock, homogeneous rate); each node is annotated for its posterior probability (in percent).

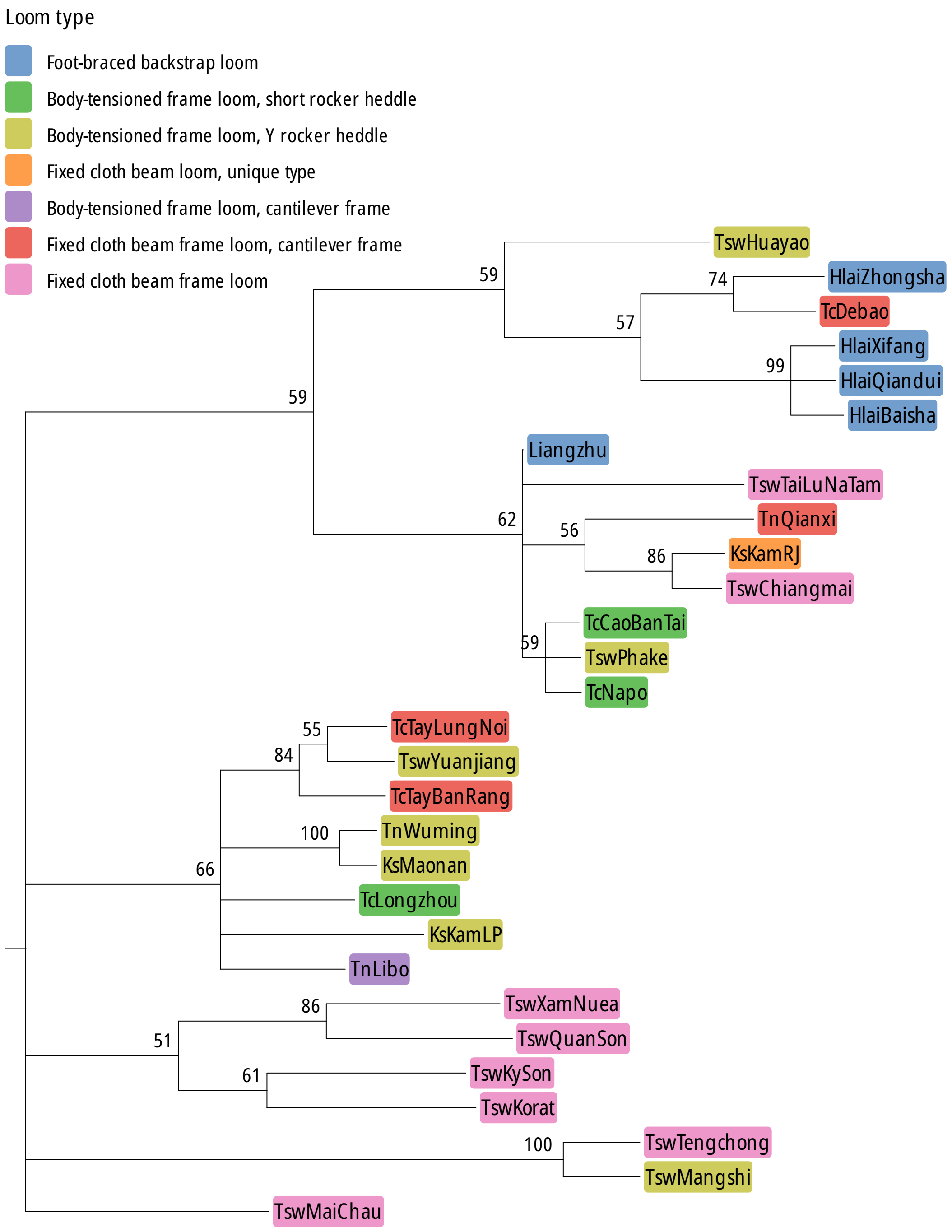

Figure S5. Majority-rule consensus tree for the looms, based on patterning characters only (binary covarion, strict clock, homogeneous rate); each node is annotated for its posterior probability (in percent).

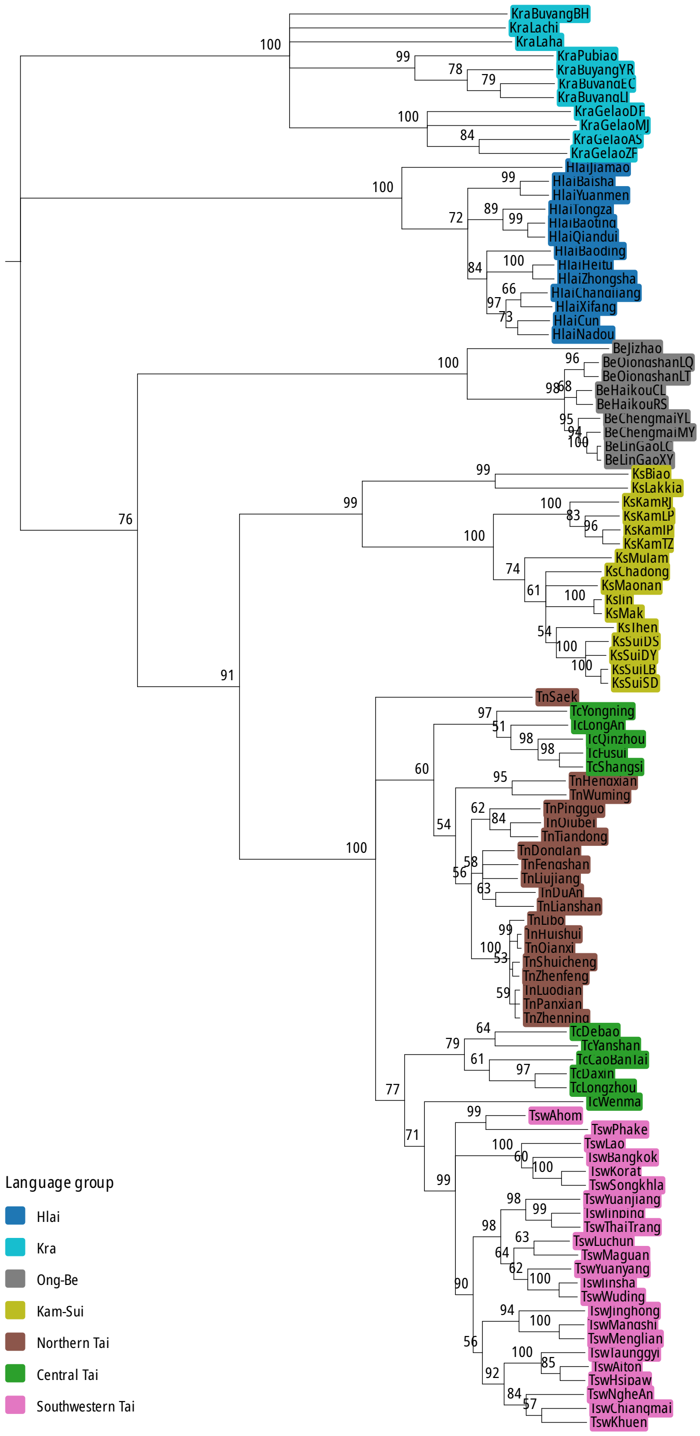

Figure S6. Majority-rule consensus tree for the languages (binary covarion, relaxed clock, heterogeneous rate by part-of-speech); each node is annotated for its posterior probability (in percent).

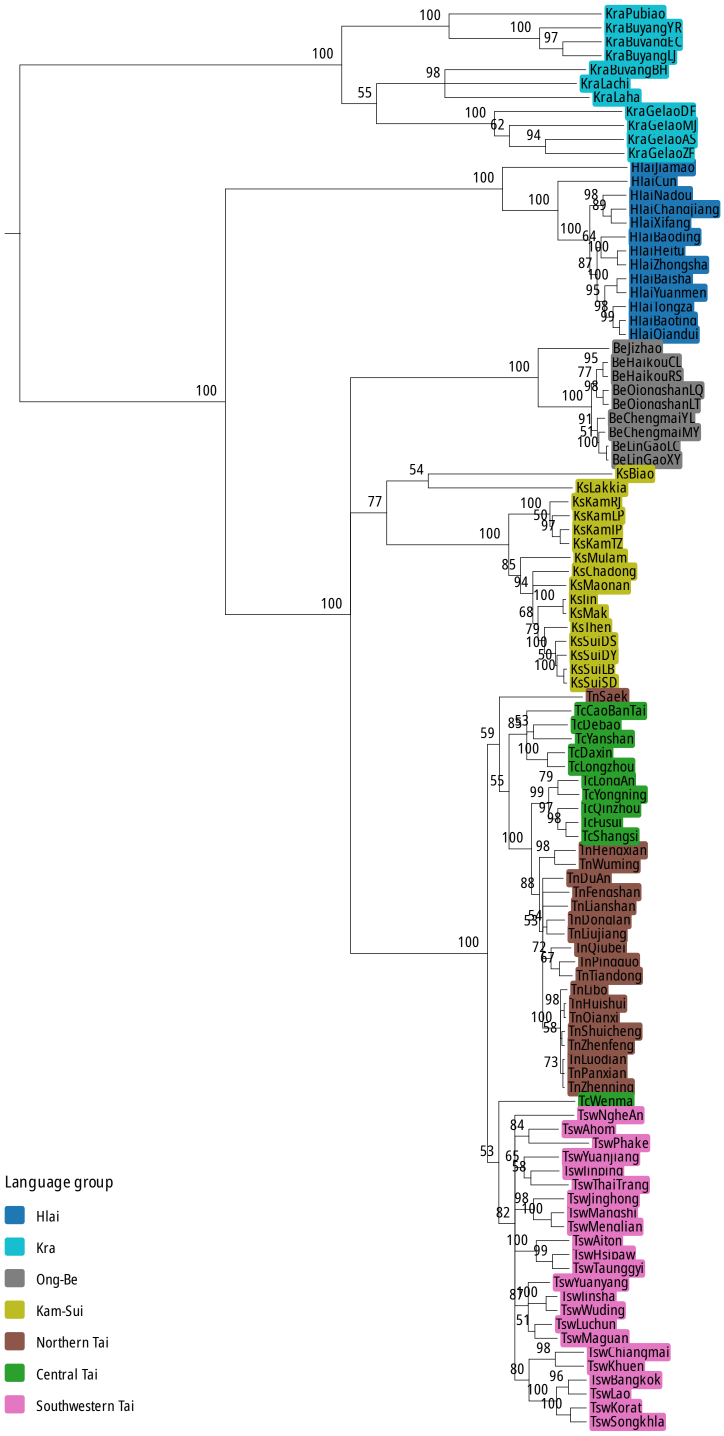

Figure S7. Majority-rule consensus tree for the languages (binary covarion, strict clock, heterogeneous rate by part-of-speech); each node is annotated for its posterior probability (in percent)

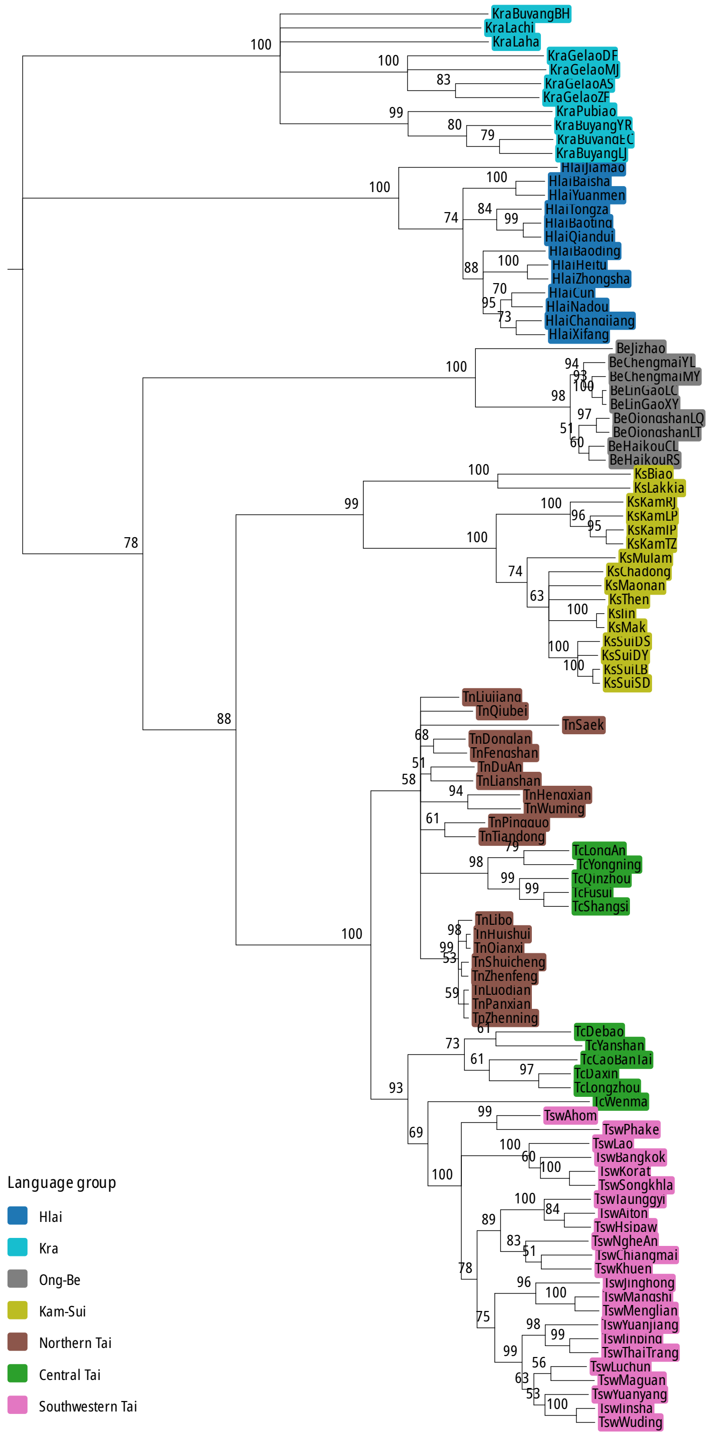

Figure S8. Majority-rule consensus tree for the languages (binary covarion, relaxed clock, homogeneous rate); each node is annotated for its posterior probability (in percent).

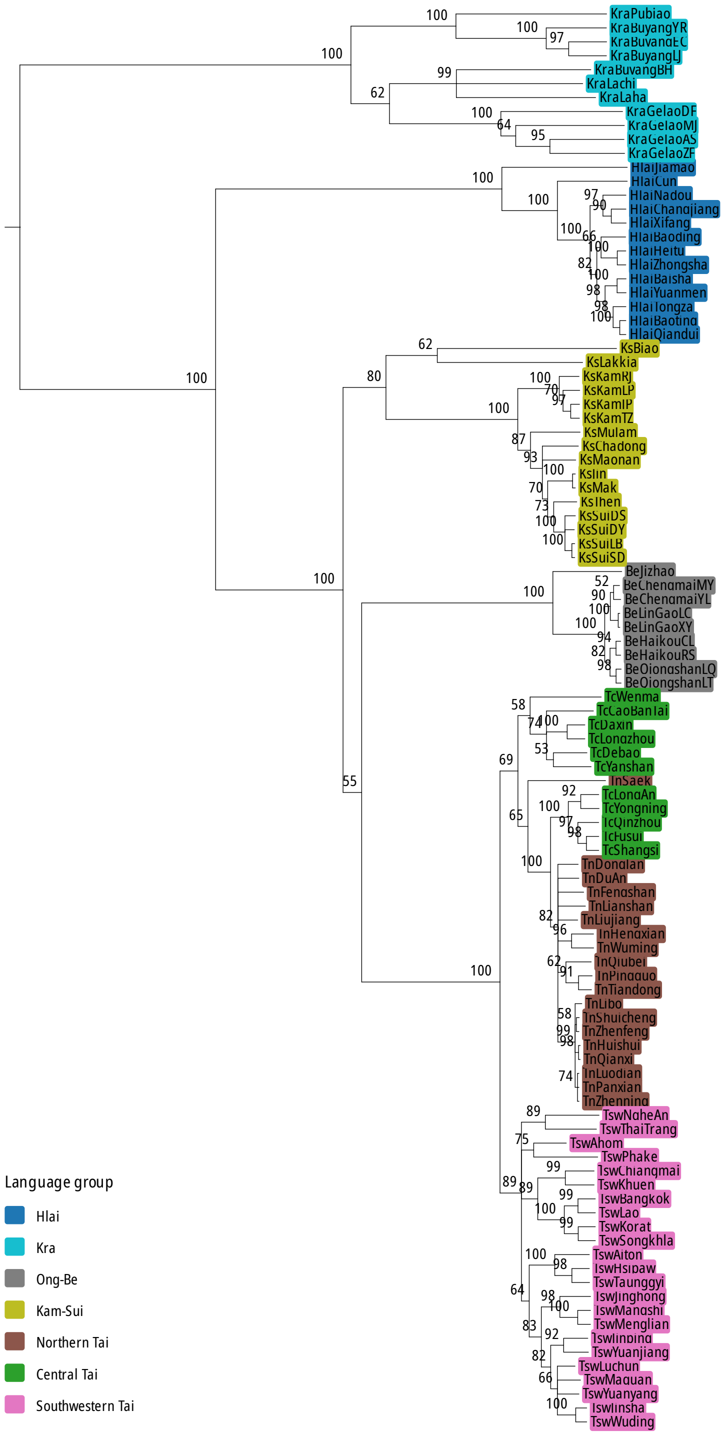

Figure S9. Majority-rule consensus tree for the languages (binary covarion, strict clock, homogeneous rate); each node is annotated for its posterior probability (in percent)

Table S2. Summary of the distribution of mutation rates for looms, by trait level (binary covarion, relaxed clock, heterogeneous rate, all traits, no weighting).

level	characters	mean	median	sd	95% HPDI
1	36	0.48	0.47	0.09	[0.3, 0.66]
2	102	0.96	0.96	0.08	[0.81, 1.12]
3	65	1.38	1.37	0.14	[1.12, 1.65]
4	13	0.84	0.77	0.32	[0.35, 1.49]

Table S3. Summary of the distribution of mutation rates for languages, by part of speech (binary covarion, relaxed clock, heterogeneous rate).

Part of speech	characters	mean	median	sd	95% HPDI
adjectives	147	1.14	1.14	0.06	[1.02, 1.27]
nouns	319	0.85	0.85	0.03	[0.78, 0.91]
verbs	138	1.15	1.15	0.07	[1.01, 1.29]
others	49	1.15	1.14	0.13	[0.93, 1.41]

Table S4. Summary of the distribution of ages for the most recent common ancestor of Kra-Dai, Kam-Tai and Tai-Yay languages (ages in millennia; binary covarion, relaxed clock, heterogeneous rate).

group	languages	mean	median	sd	95% HPDI	monophyletic
Kra-Dai	102	5.58	5.33	1.67	[2.73, 8.94]	100%
Kam-Tai	69	3.12	2.99	0.79	[1.82, 4.71]	91%
Tai-Yay	53	1.99	1.95	0.37	[1.36, 2.74]	100%

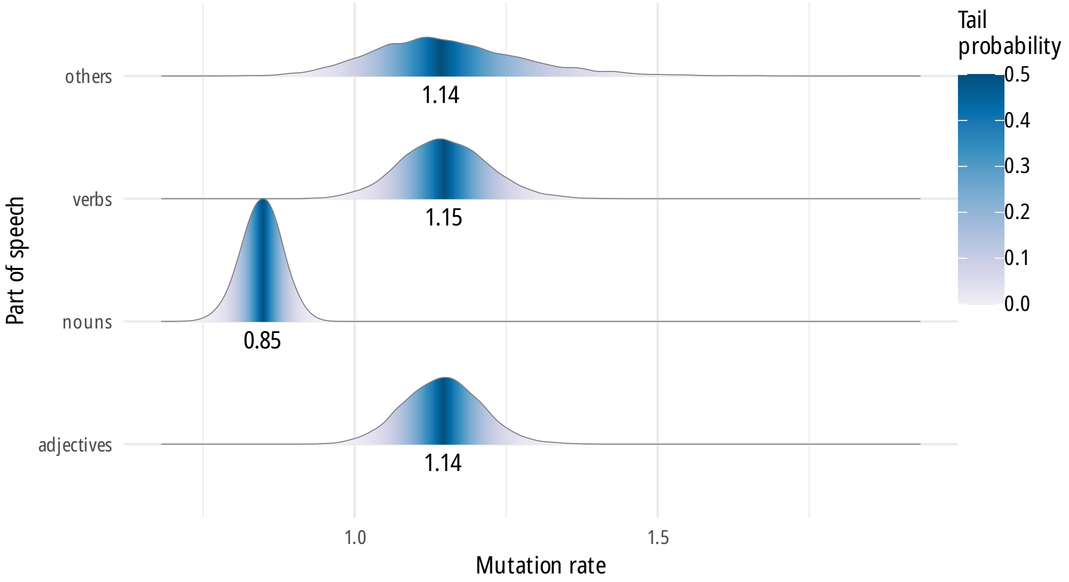

Figure S10. Probability density function and median values of the distribution of mutation rates (i.e. rates of change) in languages, by part-of-speech (binary covarion, relaxed clock, heterogeneous rate).

References

Bilderbeek, Richèl J. C. & Etienne, Rampal S. 2018. babette: BEAUti 2, BEAST2 and Tracer for R. *Methods in Ecology and Evolution* 9(9). 2034–2040. https://doi.org/10.1111/2041-210X.13032.

Blomberg, Simon P., Garland, Theodore & Ives, Anthony R. 2003. Testing for phylogenetic signal in comparative data: Behavioral traits are more labile. *Evolution* 57(4). 717–745. https://doi.org/10.1111/j.0014-3820.2003.tb00285.x.

Bouckaert, Remco, Lemey, Philippe, Dunn, Michael, Greenhill, Simon J., Alekseyenko, Alexander V., Drummond, Alexei J., Gray, Russell D., Suchard, Marc A. & Atkinson, Quentin D. 2012. Mapping the origins and expansion of the Indo-European language family. *Science* 337(6097). 957–960. https://doi.org/10.1126/science.1219669.

Bouckaert, Remco, Vaughan, Timothy G., Barido-Sottani, Joëlle, Duchêne, Sebastián, Fourment, Mathieu, Gavryushkina, Alexandra, Heled, Joseph, Jones, Graham, Kühnert, Denise, De Maio, Nicola, Matschiner, Michael, Mendes, Fábio K., Müller, Nicola F., Ogilvie, Huw A., du Plessis, Louis, Popinga, Alex, Rambaut, Andrew, Rasmussen, David, Siveroni, Igor, Suchard, Marc A., Wu, Chieh-Hsi, Xie, Dong, Zhang, Chi, Stadler, Tanja & Drummond, Alexei J. 2019. BEAST 2.5: An advanced software platform for Bayesian evolutionary analysis. *PLOS Computational Biology* 15(4). e1006650. https://doi.org/10.1371/journal.pcbi.1006650.

Drummond, Alexei J., Ho, Simon Y. W., Phillips, Matthew J. & Rambaut, Andrew. 2006. Relaxed phylogenetics and dating with confidence. *PLOS Biology* 4(5), e88. https://doi.org/10.1371/journal.pbio.0040088.

Gavryushkina, Alexandra, Welch, David, Stadler, Tanja & Drummond, Alexei J. 2014. Bayesian inference of sampled ancestor trees for epidemiology and fossil calibration. *PLOS Computational Biology* 10(12), e1003919. https://doi.org/10.1371/journal.pcbi.1003919.

Gray, Russell D. & Atkinson, Quentin D. 2003. Language-tree divergence times support the Anatolian theory of Indo-European origin. *Nature* 426(6965). 435–439. https://doi.org/10.1038/nature02029.

Lê, Sébastien, Josse, Julie & Husson, François. 2008. FactoMineR: A package for multivariate analysis. *Journal of Statistical Software* 25(1). 1–18. https://doi.org/10.18637/jss.v025.i01.

Maturana Russel, Patricio, Brewer, Brendon J., Klaere, Steffen & Bouckaert, Remco R. 2019. Model selection and parameter inference in phylogenetics using nested sampling. *Systematic Biology* 68(2). 219–233. https://doi.org/10.1093/sysbio/syy050.

Meredith, Mike & Kruschke, John. 2022. HDInterval: Highest (posterior) density intervals. Comprehensive R Archive Network. https://doi.org/10.32614/CRAN.package.HDInterval.

Morey, Stephen. 2004. The Tai languages of Assam. In Diller, Anthony V. N., Edmondson, Jerry A. & Luo, Yongxian (eds.), *The Tai-Kadai languages*, 207–253. Abingdon: Routledge.

Pebesma, Edzer. 2018. Simple features for R: Standardized support for spatial vector data. *The R Journal* 10(1). 439–446. https://doi.org/10.32614/rj-2018-009.

Penny, David, McComish, Bennet J., Charleston, Michael A. & Hendy, Michael D. 2001. Mathematical elegance with biochemical realism: The covarion model of molecular evolution. *Journal of Molecular Evolution* 53(6). 711–723. https://doi.org/10.1007/s002390010258.

Pittayaporn, Pittayawat. 2014. Layers of Chinese loanwords in Proto-Southwestern Tai as evidence for the dating of the spread of Southwestern Tai. *Manusya*: *Journal of Humanities* 17(3). 47–68. https://doi.org/10.1163/26659077-01703004.

Qin, Ling. 2013. The Liangzhu culture. In Underhill, Anne P. (ed.), *A companion to Chinese archaeology*, 574–596. Chichester: Wiley-Blackwell. https://doi.org/10.1002/9781118325698.ch28.

R Core Team. 2024. R: A language and environment for statistical computing. R Foundation for Statistical Computing. Vienna. https://www.R-project.org.

Rambaut, Andrew, Drummond, Alexei J., Xie, Dong, Baele, Guy & Suchard, Marc A. 2018. Posterior summarization in Bayesian phylogenetics using Tracer 1.7. *Systematic Biology* 67(5). 901–904. https://doi.org/10.1093/sysbio/syy032.

Revell, Liam J. 2024. phytools 2.0: An updated R ecosystem for phylogenetic comparative methods (and other things). *PeerJ* 12, e16505. https://doi.org/10.7717/peerj.16505.

Schliep, Klaus, Potts, Alastair Alastair, Morrison, David A. & Grimm, Guido W. 2016. Intertwining phylogenetic trees and networks. *PeerJ Preprints* 4, e2054v1. https://doi.org/10.7287/peerj.preprints.2054v1.

Smith, Martin R. 2019. TreeTools: Create, modify and analyse phylogenetic trees. Comprehensive R Archive Network. https://doi.org/10.5281/zenodo.3522725.

Tuffley, Chris & Steel, Mike. 1998. Modeling the covarion hypothesis of nucleotide substitution. *Mathematical Biosciences* 147(1). 63–91. https://doi.org/10.1016/S0025-5564(97)00081-3.

Wickham, Hadley, Averick, Mara, Bryan, Jennifer, Chang, Winston, McGowan, Lucy, François, Romain, Grolemund, Garrett, Hayes, Alex, Henry, Lionel, Hester, Jim, Kuhn, Max, Pedersen, Thomas, Miller, Evan, Bache, Stephan, Müller, Kirill, Ooms, Jeroen, Robinson, David, Seidel, Dana, Spinu, Vitalie, Takahashi, Kohske, Vaughan, Davis, Wilke, Claus, Woo, Kara & Yutani, Hiroaki. 2019. Welcome to the tidyverse. *Journal of Open Source Software* 4(43). 1686. https://doi.org/10.21105/joss.01686.

Yu, Guangchuang, Smith, David K., Zhu, Huachen, Guan, Yi & Lam, Tommy Tsan-Yuk. 2017. ggtree: An R package for visualization and annotation of phylogenetic trees with their covariates and other associated data. *Methods in Ecology and Evolution* 8(1). 28–36. https://doi.org/10.1111/2041-210X.12628.
